# *Cis*-eQTL analysis and functional validation of candidate susceptibility genes for high-grade serous ovarian cancer

**DOI:** 10.1038/ncomms9234

**Published:** 2015-09-22

**Authors:** Kate Lawrenson, Qiyuan Li, Siddhartha Kar, Ji-Heui Seo, Jonathan Tyrer, Tassja J. Spindler, Janet Lee, Yibu Chen, Alison Karst, Ronny Drapkin, Katja K. H. Aben, Hoda Anton-Culver, Natalia Antonenkova, Australian Ovarian Cancer Study Group, David Bowtell, Penelope M. Webb, Anna deFazio, Helen Baker, Elisa V. Bandera, Yukie Bean, Matthias W. Beckmann, Andrew Berchuck, Maria Bisogna, Line Bjorge, Natalia Bogdanova, Louise A. Brinton, Angela Brooks-Wilson, Fiona Bruinsma, Ralf Butzow, Ian G. Campbell, Karen Carty, Jenny Chang-Claude, Georgia Chenevix-Trench, Anne Chen, Zhihua Chen, Linda S. Cook, Daniel W. Cramer, Julie M. Cunningham, Cezary Cybulski, Agnieszka Dansonka-Mieszkowska, Joe Dennis, Ed Dicks, Jennifer A. Doherty, Thilo Dörk, Andreas du Bois, Matthias Dürst, Diana Eccles, Douglas T. Easton, Robert P. Edwards, Ursula Eilber, Arif B. Ekici, Peter A. Fasching, Brooke L. Fridley, Yu-Tang Gao, Aleksandra Gentry-Maharaj, Graham G. Giles, Rosalind Glasspool, Ellen L. Goode, Marc T. Goodman, Jacek Grownwald, Patricia Harrington, Philipp Harter, Hanis Nazihah Hasmad, Alexander Hein, Florian Heitz, Michelle A. T. Hildebrandt, Peter Hillemanns, Estrid Hogdall, Claus Hogdall, Satoyo Hosono, Edwin S. Iversen, Anna Jakubowska, Paul James, Allan Jensen, Bu-Tian Ji, Beth Y. Karlan, Susanne Kruger Kjaer, Linda E. Kelemen, Melissa Kellar, Joseph L. Kelley, Lambertus A. Kiemeney, Camilla Krakstad, Jolanta Kupryjanczyk, Diether Lambrechts, Sandrina Lambrechts, Nhu D. Le, Alice W. Lee, Shashi Lele, Arto Leminen, Jenny Lester, Douglas A. Levine, Dong Liang, Jolanta Lissowska, Karen Lu, Jan Lubinski, Lene Lundvall, Leon F. A. G. Massuger, Keitaro Matsuo, Valerie McGuire, John R. McLaughlin, Heli Nevanlinna, Ian McNeish, Usha Menon, Francesmary Modugno, Kirsten B. Moysich, Steven A. Narod, Lotte Nedergaard, Roberta B. Ness, Mat Adenan Noor Azmi, Kunle Odunsi, Sara H. Olson, Irene Orlow, Sandra Orsulic, Rachel Palmieri Weber, Celeste L. Pearce, Tanja Pejovic, Liisa M. Pelttari, Jennifer Permuth-Wey, Catherine M. Phelan, Malcolm C. Pike, Elizabeth M. Poole, Susan J. Ramus, Harvey A. Risch, Barry Rosen, Mary Anne Rossing, Joseph H. Rothstein, Anja Rudolph, Ingo B. Runnebaum, Iwona K. Rzepecka, Helga B. Salvesen, Joellen M. Schildkraut, Ira Schwaab, Thomas A. Sellers, Xiao-Ou Shu, Yurii B. Shvetsov, Nadeem Siddiqui, Weiva Sieh, Honglin Song, Melissa C. Southey, Lara Sucheston, Ingvild L. Tangen, Soo-Hwang Teo, Kathryn L. Terry, Pamela J. Thompson, Agnieszka Timorek, Ya-Yu Tsai, Shelley S. Tworoger, Anne M. van Altena, Els Van Nieuwenhuysen, Ignace Vergote, Robert A. Vierkant, Shan Wang-Gohrke, Christine Walsh, Nicolas Wentzensen, Alice S. Whittemore, Kristine G. Wicklund, Lynne R. Wilkens, Yin-Ling Woo, Xifeng Wu, Anna H. Wu, Hannah Yang, Wei Zheng, Argyrios Ziogas, Alvaro Monteiro, Paul D. Pharoah, Simon A. Gayther, Matthew L. Freedman

**Affiliations:** 1 Department of Preventive Medicine, Keck School of Medicine, University of Southern California Norris Comprehensive Cancer Center, Los Angeles, California 90033, USA; 2 Medical College, Xiamen University, Xiamen 361102, China; 3 Department of Medical Oncology, The Center for Functional Cancer Epigenetics, Dana-Farber Cancer Institute, Boston, Massachusetts 02215, USA; 4 Department of Oncology, Department of Public Health and Primary Care, University of Cambridge, Strangeways Research Laboratory, Cambridge CB1 8RN, UK; 5 Bioinformatics Service, Norris Medical Library, University of Southern California, Los Angeles, California 90033, USA; 6 Departments of Pathology and Medical Oncology, Dana-Farber Cancer Institute, 450 Brookline Avenue, Boston, Massachusetts 02215, USA; 7 Department for Health Evidence, Radboud University Medical Centre, PO Box 9101, 6500 HB Nijmegen, The Netherlands; 8 Comprehensive Cancer Center, The Netherlands, PO Box 19079, 3501 DB Utrecht, The Netherlands; 9 Department of Epidemiology, Director of Genetic Epidemiology Research Institute, School of Medicine, University of California Irvine, Irvine, California 92697, USA; 10 Byelorussian Institute for Oncology and Medical Radiology Aleksandrov N.N., 223040 Minsk, Belarus; 11 Cancer Prevention and Control, Rutgers Cancer Institute of New Jersey, New Brunswick, New Jersey 08903, USA; 12 Department of Obstetrics and Gynecology, Oregon Health and Science University, Portland, Oregon 97239, USA; 13 Knight Cancer Institute, Oregon Health and Science University, Portland, Oregon 97239, USA; 14 Department of Gynecology and Obstetrics, University Hospital Erlangen, Friedrich-Alexander-University Erlangen-Nuremberg, Comprehensive Cancer Center Erlangen-EMN, 91054 Erlangen, Germany; 15 Department of Obstetrics and Gynecology, Duke University Medical Center, Durham, North Carolina 27710, USA; 16 Gynecology Service, Department of Surgery, Memorial Sloan Kettering Cancer Center, New York, New York 10065, USA; 17 Department of Gynecology and Obstetrics, Haukeland University Hospital, N-5058 Bergen, Norway; 18 Centre for Cancer Biomarkers, Department of Clinical Science, University of Bergen, N-5020 Bergen, Norway; 19 Gynaecology Research Unit, Hannover Medical School, 30625 Hannover, Germany; 20 Division of Cancer Epidemiology and Genetics, National Cancer Institute, Bethesda Maryland, 20892, USA; 21 Canada’s Michael Smith Genome Sciences Centre, BC Cancer Agency, Vancouver, British Columbia, Canada V5Z 1L3; 22 Department of Biomedical Physiology and Kinesiology, Simon Fraser University, Burnaby, British Columbia, Canada V5A 1S6; 23 Cancer Epidemiology Centre, Cancer Council Victoria, Melbourne, Victoria 3004, Australia; 24 Department of Obstetrics and Gynecology, University of Helsinki and Helsinki University Central Hospital, 00100 Helsinki, Finland; 25 Department of Pathology, Helsinki University Central Hospital, FI-00014 Helsinki, Finland; 26 Cancer Genetics Laboratory, Research Division, Peter MacCallum Cancer Centre, Melbourne, Victoria 3002, Australia; 27 Department of Pathology, University of Melbourne, Parkville, Victoria 3010, Australia; 28 Sir Peter MacCallum Department of Oncology, University of Melbourne, Parkville, Victoria 3010, Australia; 29 Cancer Research UK Clinical Trials Unit, The Beatson West of Scotland Cancer Centre, Glasgow G12 0YN, UK; 30 German Cancer Research Center (DKFZ), Division of Cancer Epidemiology, 69120 Heidelberg, Germany; 31 Cancer Division, QIMR Berghofer Medical Research Institute, Brisbane, Queensland 4006, Australia; 32 Department of Biostatistics, Moffitt Cancer Center, Tampa, Florida 33612, USA; 33 Division of Epidemiology and Biostatistics, Department of Internal Medicine, University of New Mexico, Albuquerque, New Mexico 87131, USA; 34 Harvard School of Public Health, Boston, Massachusetts 02215, USA; 35 Obstetrics and Gynecology Epidemiology Center, Brigham and Women’s Hospital and Harvard Medical School, Boston, Massachusetts 02215, USA; 36 Department of Laboratory Medicine and Pathology, Mayo Clinic, Rochester, Minnesota 55905, USA; 37 Department of Genetics and Pathology, Pomeranian Medical University, Szczecin, Poland; 38 Centre for Cancer Genetic Epidemiology, Department of Public Health and Primary Care, University of Cambridge, Cambridge CB1 8RN, UK; 39 Department of Epidemiology, The Geisel School of Medicine at Dartmouth, Lebanon, New Hampshire 03756, USA; 40 Department of Gynecology and Gynecologic Oncology, Kliniken Essen-Mitte, 45136 Essen, Germany; 41 Department of Gynecology and Gynecologic Oncology, Dr Horst Schmidt Kliniken Wiesbaden, 65199 Wiesbaden, Germany; 42 Department of Gynecology, Jena University Hospital—Friedrich Schiller University, 07737 Jena, Germany; 43 Faculty of Medicine, University of Southampton, Southampton SO16 5YA, UK; 44 Department of Obstetrics, Gynecology and Reproductive Sciences, University of Pittsburgh School of Medicine, Pittsburgh, Pennsylvania 15213, USA; 45 Ovarian Cancer Center of Excellence, University of Pittsburgh, Pittsburgh, Pennsylvania, 15213, USA; 46 University Hospital Erlangen, Institute of Human Genetics, Friedrich-Alexander-University Erlangen-Nuremberg, 91054 Erlangen, Germany; 47 Division of Hematology and Oncology, Department of Medicine, David Geffen School of Medicine, University of California at Los Angeles, Los Angeles California 90095, USA; 48 Biostatistics and Informatics Shared Resource, University of Kansas Medical Center, Kansas City, Kansas, 66160, USA; 49 Shanghai Cancer Institute, Shanghai 200030, China; 50 Department of Women’s Cancer, Institute for Women’s Health, University College London, London W1T 7DN, UK; 51 Centre for Epidemiology and Biostatistics, Melbourne School of Population and Global Health, The University of Melbourne, Melbourne, Victoria 3010, Australia; 52 Department of Health Science Research, Mayo Clinic, Rochester, Minnesota 55905, USA; 53 Cancer Prevention and Control, Samuel Oschin Comprehensive Cancer Institute, Cedars-Sinai Medical Center, Los Angeles, California 90048, USA; 54 Community and Population Health Research Institute, Department of Biomedical Sciences, Cedars-Sinai Medical Center, Los Angeles, California 90048, USA; 55 Cancer Research Initiatives Foundation, Sime Darby Medical Centre, 47500 Subang Jaya, Malaysia; 56 Department of Epidemiology, The University of Texas MD Anderson Cancer Center, Houston, Texas 77030, USA; 57 Departments of Obstetrics and Gynaecology, Hannover Medical School, 30625 Hannover, Germany; 58 Institute of Cancer Epidemiology, Danish Cancer Society, DK-2100 Copenhagen, Denmark; 59 Molecular Unit, Department of Pathology, Herlev Hospital, University of Copenhagen, 1165 Copenhagen, Denmark; 60 Gyn Clinic, Rigshospitalet, University of Copenhagen, 2100 Copenhagen, Denmark; 61 Division of Epidemiology and Prevention, Aichi Cancer Center Research Institute, Nagoya 464-0021, Japan; 62 Department of Statistical Science, Duke University, Durham, North Carolina 27708, USA; 63 Department of Gynecology, Rigshospitalet, University of Copenhagen, 2100 Copenhagen, Denmark; 64 Women’s Cancer Program at the Samuel Oschin Comprehensive Cancer Institute, Cedars-Sinai Medical Center, Los Angeles, California 90048, USA; 65 Department of Virus, Lifestyle and Genes, Danish Cancer Society Research Center, 2100 Copenhagen, Denmark; 66 Department of Gynaecology, The Juliane Marie Centre, Rigshospitalet, University of Copenhagen, 2100 Copenhagen, Denmark; 67 Department of Public Health Sciences, College of Medicine, Medical University of South Carolina, Charleston, South Carolina 29435, USA; 68 Radboud University Medical Mentre, Radboud Institute for Health Sciences, 6500 HB Nijmegen, The Netherlands; 69 Vesalius Research Center, VIB, 3000 Leuven, Belgium; 70 Laboratory for Translational Genetics, Department of Oncology, University of Leuven, B-3000 Leuven, Belgium; 71 Division of Gynecological Oncology, Department of Oncology, University Hospitals Leuven, B-3000 Leuven, Belgium; 72 Cancer Control Research, BC Cancer Agency, Vancouver, British Columbia, Canada V5Z 1L3; 73 Department of Cancer Prevention and Control, Roswell Park Cancer Institute, Buffalo, New York 14263, USA; 74 College of Pharmacy and Health Sciences, Texas Southern University, Houston, Texas 77004, USA; 75 Department of Cancer Epidemiology and Prevention, Maria Sklodowska-Curie Memorial Cancer Center and Institute of Oncology, Warsaw, Poland; 76 Department of Gynecologic Oncology, The University of Texas MD Anderson Cancer Center, Houston, Texas 77030, USA; 77 Department of Gynaecology, Radboud University Medical Centre, 6500 HB Nijmegen, The Netherlands; 78 Department of Preventive Medicine, Kyushu University Faculty of Medical Sciences, 819-0395 Fukuoka, Japan; 79 Department of Health Research and Policy - Epidemiology, Stanford University School of Medicine, Stanford California 94305, USA; 80 Prosserman Centre for Health Research, Lunenfeld-Tanenbaum Research Institute, Mount Sinai Hospital, Toronto, Ontario, Canada M5G 1X5; 81 Institute of Cancer Sciences, Wolfson Wohl Cancer Research Centre, Beatson Institute for Cancer Research, University of Glasgow, Glasgow G61 1QH, UK; 82 Women’s Cancer Research Program, Magee-Women’s Research Institute and University of Pittsburgh Cancer Institute, Pittsburgh, Pennsylvania 15213, USA; 83 Department of Epidemiology, University of Pittsburgh Graduate School of Public Health, Pittsburgh, Pennsylvania 15261, USA; 84 Department of Epidemiology and Biostatistics, Memorial Sloan Kettering Cancer Center, New York, New York 10017, USA; 85 Department of Pathology, Rigshospitalet, University of Copenhagen, 2100 Copenhagen, Denmark; 86 The University of Texas School of Public Health, Houston, Texas 77030, USA; 87 Department of Obstetrics and Gynaecology, University Malaya Medical Centre, University Malaya, 50603 Kuala Lumpur, Malaysia; 88 Department of Gynecological Oncology, Roswell Park Cancer Institute, Buffalo, New York 14263, USA; 89 Department of Community and Family Medicine, Duke University Medical Center, Durham, North Carolina 27710, USA; 90 Department of Cancer Epidemiology, Moffitt Cancer Center, Tampa, Florida 33612, USA; 91 Channing Division of Network Medicine, Brigham and Women’s Hospital and Harvard Medical School, Boston, Massachusetts 02215, USA; 92 Department of Epidemiology, Harvard T.H. Chan School of Public Health, Boston, Massachusetts 02215, USA; 93 Department of Chronic Disease Epidemiology, Yale School of Public Health, New Haven, Connecticut 06510, USA; 94 Department of Gynecologic-Oncology, Princess Margaret Hospital, and Department of Obstetrics and Gynecology, Faculty of Medicine, University of Toronto, Toronto, Ontario, Canada M5S 2J7; 95 Program in Epidemiology, Division of Public Health Sciences, Fred Hutchinson Cancer Research Center, Seattle, Washington 98109, USA; 96 Department of Epidemiology, University of Washington, Seattle, Washington 98195, USA; 97 Cancer Control and Population Sciences, Duke Cancer Institute, Durham, North Carolina 27710, USA; 98 Institut für Humangenetik Wiesbaden, 65187 Wiesbaden, Germany; 99 Division of Epidemiology, Department of Medicine, Vanderbilt Epidemiology Center and Vanderbilt-Ingram Cancer Center, Vanderbilt University School of Medicine, Nashville, Tennessee 37232, USA; 100 Cancer Epidemiology Program, University of Hawaii Cancer Center, Honolulu, Hawaii, 96813, USA; 101 Department of Gynaecological Oncology, Glasgow Royal Infirmary, Glasgow G4 0SF, UK; 102 University Malaya Cancer Research Institute, Faculty of Medicine, University Malaya Medical Centre, University Malaya, 50603 Kuala Lumpur, Malaysia; 103 Department of Obstetrics, Gynecology and Oncology, IInd Faculty of Medicine, Warsaw Medical University and Brodnowski Hospital, Warsaw, Poland; 104 Department of Obstetrics and Gynecology, University of Ulm, 89075 Ulm, Germany; 105 Cancer Epidemiology Program, Division of Population Sciences, H. Lee Moffitt Cancer Center & Research Institute, Tampa, Florida 33612, USA; 106 Peter MacCallum Cancer Centre, Melbourne, Victoria 3002, Australia; 107 QIMR Berghofer, Brisbane, Queensland 4006, Australia; 108 Westmead Hospital and Center for Cancer Research, University of Sydney at Westmead Millennium Institute, Westmead, Sydney, New South Wales 2145, Australia

## Abstract

Genome-wide association studies have reported 11 regions conferring risk of high-grade serous epithelial ovarian cancer (HGSOC). Expression quantitative trait locus (eQTL) analyses can identify candidate susceptibility genes at risk loci. Here we evaluate *cis*-eQTL associations at 47 regions associated with HGSOC risk (*P*≤10^−5^). For three *cis*-eQTL associations (*P*<1.4 × 10^−3^, FDR<0.05) at 1p36 (*CDC42*), 1p34 (*CDCA8*) and 2q31 (*HOXD9*), we evaluate the functional role of each candidate by perturbing expression of each gene in HGSOC precursor cells. Overexpression of HOXD9 increases anchorage-independent growth, shortens population-doubling time and reduces contact inhibition. Chromosome conformation capture identifies an interaction between rs2857532 and the *HOXD9* promoter, suggesting this SNP is a leading causal variant. Transcriptomic profiling after HOXD9 overexpression reveals enrichment of HGSOC risk variants within HOXD9 target genes (*P*=6 × 10^−10^ for risk variants (*P*<10^−4^) within 10 kb of a HOXD9 target gene in ovarian cells), suggesting a broader role for this network in genetic susceptibility to HGSOC.

## Abstract (web summary)

Genome-wide association studies have identified regions which confer risk of high-grade serous epithelial ovarian cancer. Here the authors use expression quantitative train locus analysis to identify candidate genes and functionally characterise them, identifying a role for HOXD9 in ovarian cancer.

## Graphical abstract

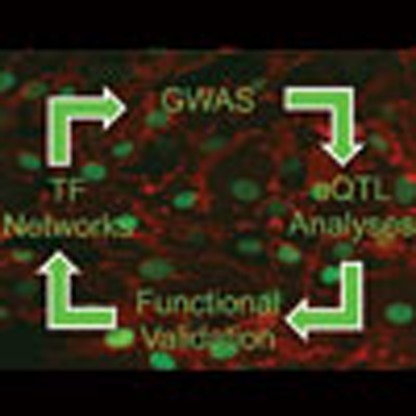

Genome-wide association studies (GWAS) have identified hundreds of common single nucleotide polymorphisms (SNPs) associated with cancer predisposition. However, the functional role of these genetic risk variants in disease biology and the target cancer susceptibility genes have been described for only a handful of risk regions^1^,^2^,^3^,^4^,^5^. Approximately 90% of risk-associated alleles lie within non-protein coding regions of the genome, suggesting that some reside within regulatory elements that influence the expression of target genes. In support of this, common risk variants often coincide with regulatory biofeatures, including transcription factor-binding sites and regions of active chromatin, such as transcriptional enhancers^4^,^6^,^7^,^8^.

Epithelial ovarian cancer (EOC) has a major heritable component, a proportion of which is due to common low-penetrance-susceptibility alleles. High-grade serous ovarian cancer (HGSOC) accounts for about 60% of all invasive EOC cases. Eleven common variant risk loci have so far been identified HGSOC using GWAS and replication analyses^5^,^9^,^10^,^11^,^12^,^13^,^14^. While it is estimated that hundreds of additional risk variants are likely to exist, their identification in the future will be challenging because of the limitations in sample size restricting the power to detect genetic associations at genome-wide levels of significance. One approach to identify additional HGSOC risk alleles may be to use biological and functional information to provide additional evidence for risk associations in regions that are sub-genome-wide significant in genetic association studies.

Expression quantitative trait locus (eQTL) analysis is a straightforward approach to the identification of candidate susceptibility genes at risk loci. The goal is to identify allelic variants associated with gene expression on the basis that a proportion of transcripts are under genetic control. A transcript that is correlated with a risk variant in a relevant tissue or cell type represents a strong candidate susceptibility gene. EQTL analyses have recently identified candidate susceptibility genes for multiple cancer types including breast, prostate, lung and colorectal cancers^3^,^15^,^16^,^17^. However, rarely have functional studies been performed to validate the role of these candidate genes.

In the current study, we evaluate whether eQTL analysis performed in primary HGSOCs can identify candidate ovarian cancer susceptibility genes at genomic regions showing evidence of susceptibility to HGSOC (*P* value for association <1 × 10^−5^). We aimed to establish if eQTL analyses could provide additional biological evidence supporting putative susceptibility loci that have so far failed to reach genome-wide significance. Having identified significant *cis*-eQTL associations, we evaluate the role of candidate genes in the early stage development of HGSOC through targeted perturbation of candidate gene expression in two HGSOC precursor cell types and use chromosome conformation capture assays to identify physical interactions between a target gene and risk-associated SNPs. Finally, we use transcriptomic profiling to identify downstream targets of validated susceptibility genes, to identify common biological pathways associated with neoplastic development, and to provide functional evidence supporting additional potential HGSOC susceptibility loci.

## Results

### Risk-associated variants in high-grade serous ovarian cancer

Genetic association analyses were performed using data from the Ovarian Cancer Association Consortium (OCAC) case–control studies^5^,^9^,^10^,^11^,^12^,^13^,^14^. Genotype data were available for 15,397 women of European ancestry, diagnosed with invasive epithelial EOC, 9,608 of whom were diagnosed with serous EOC and 30,816 controls. These were from 43 studies from 11 countries that were part of several GWAS and the Collaborative Oncological Gene-environment Study (COGS) genotyping project^9^,^18^,^19^. A meta-analysis of these data identified 47 susceptibility regions associated with HGSOC risk at a statistical threshold of *P*<10^−5^ (Supplementary Table 1). Eleven of these risk loci reached genome-wide levels of significance (*P*≤5 × 10^−8^) (refs 5, 9, 10, 11, 12, 13, 14).

### Identifying *cis*-eQTL associations at HGSOC risk loci

Using profiles of gene expression, somatic copy number variation and methylation available for 339 primary HGSOCs from The Cancer Genome Atlas (TCGA) project, we evaluated determinants of gene expression in ovarian cancer. Copy number variation explains 14% and methylation 4.1% of variation in gene expression. We then measured the contribution of *cis*-expression quantitative trait loci, adjusting for somatic copy number variation and CpG methylation as previously described^15^. For these analyses we defined *cis*- as a 250-kb region spanning each SNP. The *cis*-eQTL-based analysis explained a further 0.25% of the variation in gene expression in HGSOCs. From 906,600 variants on the Affymetrix SNP6.0 arrays, this represents 592 eQTL associations with a false discovery rate (FDR) <0.1.

**Figure 1:**
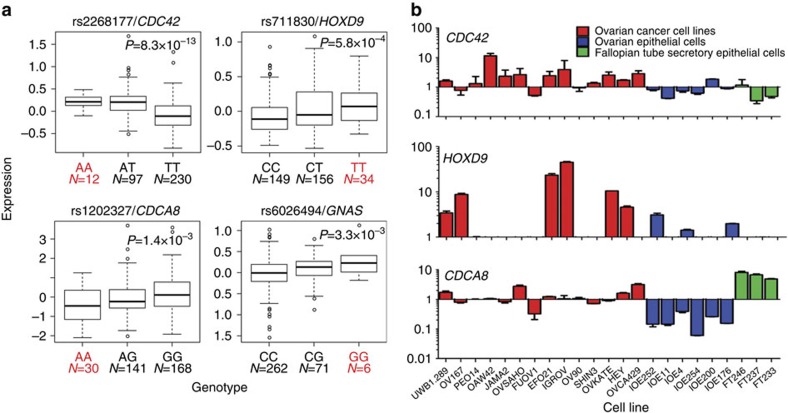
EQTL analyses identify candidate genes at HGSOC risk loci. (**a**) *CDC42* at 1p36, *HOXD9* at 2q31, *CDCA8* at 1p34 and *GNAS* at 20q13. Genotypes associated with increased risk are indicated in red font. On the boxplots the horizontal line indicates the median, the box indicates the first to third quartile of expression and whiskers indicate 1.5 × the interquartile range. (**b**) Analysis of the expression of three genome-wide significant genes in 14 ovarian cancer cell lines (predominantly of high-grade serous histology), six TERT-immortalized ovarian epithelial (IOE) cell lines and three TERT, shRNA-p53 and mutant CDK4 immortalized fallopian tube (FT) epithelial cell lines.

Next, we restricted our analyses to SNPs located at the 47 HGSOC risk loci (*P*<10^−5^). We identified four statistically significant eQTL associations: these associations were between rs711830 and *HOXD9* at 2q31 (*P*=5.8 × 10^−4^, FDR=0.03, Wald test); rs2268177 and *CDC42* at 1p36 (*P*=8.4 × 10^−13^, FDR=9.1 × 10^−11^, Wald test); rs12023270 and *CDCA8* at 1p34 (*P*=1.4 × 10^−3^, FDR=0.05, Wald test); and rs6026496 and *GNAS* at 20q13 (*P*=3.3 × 10^−3^, FDR=0.09, Wald test). Of these, only rs711830 at 2q31 locus is associated with HGSOC at genome-wide significance (*P*=9.0 × 10^−14^). For the remaining three loci the associations were borderline genome-wide significant: *P*=6.8 × 10^−7^ at 1p36, *P*=1.4 × 10^−7^ at 1p34 and *P*=5.1 × 10^−7^ at 20q21. These data are summarized in Fig. 1a and Table 1.

**Figure 2:**
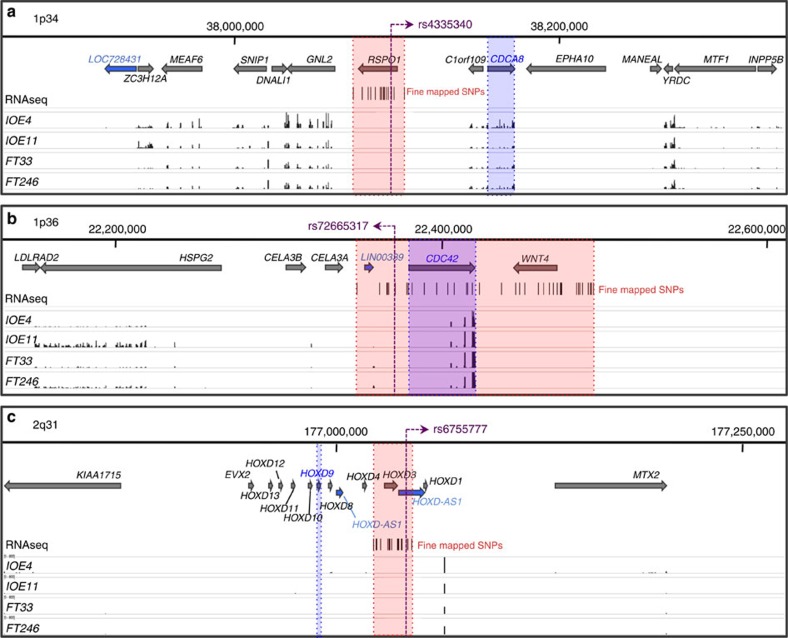
Fine mapped HGSOC risk regions and gene expression in HGSOC precursor cells. A 0.5-Mb region spanning each risk locus is shown. The region defined by fine mapping is indicated by a red box, the candidate gene outlined by a blue box and candidate genes identified by eQTL analyses are indicated in bold blue font. The most significant SNP is indicated by a purple dashed line. RNAseq data for HGSOC precursor cells are shown. (**a**) At the 1p34 locus, the risk SNPs cluster around the *RSPO1* gene, but this gene is not expressed in IOE and fallopian tube (FT) cells. (**b**) At 1p36, the risk SNPs span a 145-kb window encompassing *LIN00339, CDC42* and *WNT4*. (**c**) At 2q31, the 19 risk SNPs cluster around *HOXD3*, ∼45kb telomeric to *HOXD9*.

Using quantitative PCR with reverse transcription (RT–qPCR) analysis we quantified expression of *HOXD9*, *CDC42* and *CDCA8* in ovarian cancer cell lines (*N*=14) and ovarian (*N*=6) and fallopian (*N*=3) epithelial cells (Fig. 1b). *CDC42* was expressed in all samples with highest expression levels observed in cancer cell lines (*P*<0.028). *HOXD9* expression was detected in ∼80% of ovarian cancer cell lines and all normal ovarian epithelial cell lines, but was absent in the normal fallopian tube epithelial cell lines. *CDCA8* was expressed by all three cell types, and was significantly lower in ovarian epithelial cells compared with ovarian cancer cells (*P*=5.0 × 10^−4^) and fallopian epithelial cells (*P*=2.0 × 10^−3^). Figure 2 illustrates each genomic region, the location of all candidate functional SNPs and the expression of all of the genes in the region profiled in four ovarian cancer precursor cell lines using RNA sequencing.

### Functional validation of candidate susceptibility genes

We evaluated the functional effects of perturbing the expression of the top three *cis*-eQTL target genes (FDR<0.05)—*CDC42*, *CDCA8* and *HOXD9*—in cell line models of the early stages of neoplastic transformation of HGSOC. Each gene was evaluated in the two cell types that are proposed to be the precursors of HGSOC; fallopian tube secretory epithelial cells and ovarian surface epithelial cells. Both cell lines were engineered to be deficient in p53 signalling, since this event occurs in almost all HGSOCs^20^,^21^. Fallopian tube cells were immortalized by expression of *TERT* followed by short hairpin RNA (shRNA)-mediated knockdown of p53 and expression of the CDK4^R24C^ inhibition-resistant mutant CDK4 allele (FT246-shp53-R24C)^22^. Ovarian surface epithelial cells were immortalized with *TERT* alone^23^ after which we generated a p53-deficient model by stably expressing a dominant negative p53 allele (IOE11-DNp53). In the latter model, loss of functional p53 signalling was confirmed using *in vitro* assays: upregulation of p21 following exposure to ionizing radiation was attenuated, and population-doubling times were reduced in cells expressing the DNp53 construct (Supplementary Fig. 1).

**Figure 3:**
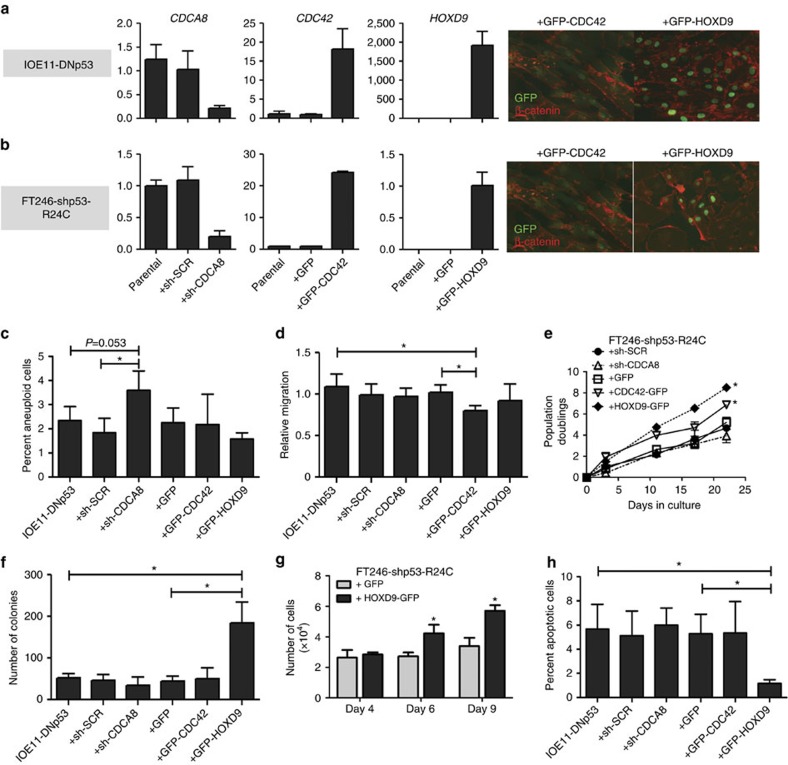
Characterization of overexpression and knockdown models of eQTL genes. ShRNAs targeting *CDCA8* were used to knockdown *CDCA8* expression and C-terminal GFP fusion proteins of *CDC42* and *HOXD9* were delivered by lentiviral transduction to overexpress these two genes in (**a**) IOE11-DNp53 cells and (**b**) FT246-shp53-R24C cells. (left panels) Gene expression measured by RT–qPCR; (right panels) protein expression visualized by fluorescence microscopy, CDC42 expression is detected throughout the cell, whereas HOXD9 expression is exclusively nuclear. (**c**) Quantification of aneuploid cell population (>4N) following perturbation of each gene, in IOE11-DNp53 models. (**d**) Overexpression of CDC42 is associated with reduced migration in IOE-DNp53. (**e**) Growth curve analysis of anchorage-dependent growth, cells expressing CDC42 and HOXD9 have significantly shorter population-doubling times. (**f**) Overexpression of HOXD9 is associated with increased colony formation in anchorage-independent growth assays in IOE11-DNp53. (**g**) Contact inhibition assay, HOXD9-expressing FT246-shp53-R24C cells are more proliferative under conditions of high cell density, compared with GFP-expressing controls. (**h**) Overexpression of HOXD9 is associated with reduced apoptosis. Data shown represent mean±s.d. of three independent experiments. *P<0.05, two-tailed paired *t*-test.

For each cell type, we created isogenic models of candidate gene overexpression or knockdown, mimicking the trends in expression associated with the risk allele as defined by the eQTL associations. Thus, we stably overexpressed *CDC42* and *HOXD9* as C-terminal green fluorescent protein (GFP) fusion proteins, and downregulated *CDCA8* using pooled targeting shRNAs. Overexpression or knockdown of each gene was confirmed by RT–qPCR (Fig. 3a(i)). We confirmed expression of the fusion proteins for CDC42 and HOXD9 by fluorescence microscopy (Fig. 3a(ii)). CDC42 was detected throughout the cell, whereas HOXD9 expression was restricted to the nucleus. We then evaluated the engineered cell lines for phenotypes that are indicative of neoplastic transformation and tumour development, specifically anchorage-dependent and -independent growth, migration, invasion, apoptosis and DNA content (ploidy). The results of these analyses are shown in Fig. 3c–h.

### Effects of CDCA8 downregulation

Using lentiviral delivery of *CDCA8*-targeting shRNAs, *CDCA8* gene expression was knocked down by 78% in IOE11-DNp53 cells, and 85% in FT246-shp53-R24C cell lines compared with parental cells and cell lines expressing a non-targeting, scrambled (SCR) shRNA (IOE11-DNp53-shSCR and FT246-shp53-R24C-shSCR). Downregulation of *CDCA8* had no significant effect on anchorage-dependent or -independent growth, invasion or migration in either IOE11-DNp53 or FT246-shp53-R24C cells. However, using propidium iodide staining we observed a 2.2-fold increase in the proportion of aneuploid cells in IOE11-DNp53-shCDCA8 cultures compared with IOE11-DNp53-shSCR controls (*P*=0.026, two-tailed paired *t*-test) (Fig. 3c).

### Effects of CDC42 overexpression

IOE11-DNp53 and FT246-shp53-R24C engineered to overexpress *CDC42* showed 18- and 24-fold increase in *CDC42* expression, respectively, compared with non-transduced and GFP-transduced control cell lines (IOE11-DNp53-GFP and FT246-shp53-R24C-GFP). Overexpression of *CDC42* was associated with a 20% reduction in migration (*P*=0.040) compared with IOE11-DNp53-GFP and IOE11-DNp53 control cells (Fig. 3d) but no other cellular phenotypes were affected in this model. However, FT246-shp53-R24C-CDC42 cells had significantly shorter population-doubling times in anchorage-dependent growth assays (Fig. 3e).

### Effects of HOXD9 overexpression

*HOXD9* expression was undetectable in IOE11-DNp53 and FT246-shp53-R24C cells and GFP-transduced cells; but after lentiviral infection of a *HOXD9* construct, IOE11-DNp53 cells and FT246-shp53-R24C cells showed robust *HOXD9* expression. IOE11-DNp53-HOXD9 cells demonstrated a 4.2-fold increase in anchorage-independent growth relative to parental cells and control cells expressing GFP only (*P*=0.026, two-tailed paired *t*-test, Fig. 3f). FT246-shp53-R24C-HOXD9 cells exhibited significantly shorter population-doubling times than control cells (Fig. 3e), and by light microscopy, we observed that HOXD9-expressing cells tended to become more tightly packed into the monolayer. We therefore performed contact inhibition assays, which revealed that these cells were more proliferative under conditions of high cell density, compared with control FT246-shp53-R24C-GFP cells (Fig. 3g). Finally, cell cycle analyses in diploid IOE11-DNp53-HOXD9 cells showed a ∼78% reduction in the proportion of apoptotic cells relative to GFP-expressing controls (*P*=0.034, two-tailed paired *t*-test, Fig. 3h).

### Interactions between 2q31 risk SNPs and *HOXD9*

**Figure 4:**
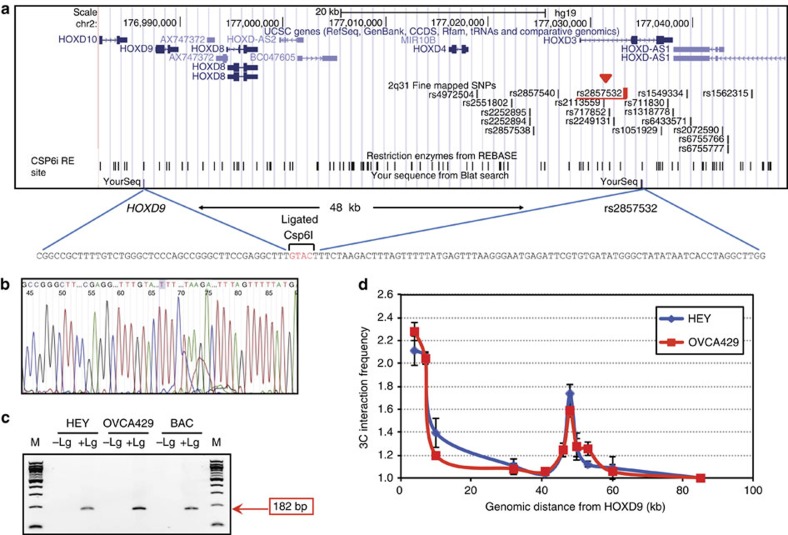
3C Analysis at the 2q31 locus. We systematically tested for interactions between the *HOXD9* promoter and risk SNPs. We identified an interaction between a region containing rs2857532 and the *HOXD9* promoter. (**a**) Map of the genomic region, showing the *HOXD* gene cluster and the fine mapped risk SNPs. (**b**) The interaction was verified by sequencing. (**c**) Agarose gel electrophoresis of ligation products. There was no ligation product in the absence of ligase (Lg). M, 100-bp molecular weight marker. (**d**) Quantification of 3C interaction frequencies between a constant fragment containing the *HOXD9* promoter and each target fragment. In both cell lines, a peak of interaction is observed with the fragment containing the rs2857532 variant located 48 kb away from the constant fragment. The *y* axis refers to semi-quantitative PCR products from 3C libraries in both cell lines normalized by each interrogated ligation PCR product using BAC control template. The error bars represent the s.e.m.

Because of the strong neoplastic phenotypes associated with overexpression of HOXD9, we evaluated the 2q31 locus in more detail. While the SNP with the strongest association is the most obvious candidate for being the causal variant in this region, other correlated SNPs with slightly weaker associations may be the true causal variant. On the basis of a comparison of the log likelihoods from the association testing for each SNP with the most significant SNP there are 19 SNPs that are candidates for being the causal variant at odds of 100:1 or better (Fig. 4). We created a chromosome conformation capture (3C) interaction map of the region, systematically testing for interactions between the *HOXD9* promoter (anchor) and 11 restriction fragments covering the 19 risk SNPs (targets). We observed an interaction between the region containing rs2857532 and the *HOXD9* promoter in two different epithelial ovarian cancer cell lines (Fig. 4). There was no evidence of interaction between the *HOXD9* promoter and any of the other 18 risk-associated variants at this locus. Using the Match algorithm and TRANSFAC matrices we identified transcription factors that differentially bind to the reference (A) and alternative (G) alleles of the rs2857532 variant. The alternative allele creates a binding site for HOMEZ, BEN and RelA-p65 transcription factors (Table 2). Analysis of TCGA data confirmed that these three transcription factors are expressed in HGSOC. These transcription factors do not bind the reference allele and thus represent candidate transcription factors that may function upstream of rs2857532 to modulate *HOXD9* expression during ovarian cancer development.

### Downstream targets of HOXD9

RNA sequencing was used to profile transcriptomic changes resulting from HOXD9 overexpression in IOE11-DNp53 and FT246-shp53-R24C cells; expression of 10 target genes was validated by RT–qPCR (Supplementary Fig. 2). Transcriptional networks downstream of risk-associated genes have themselves been shown to regulate germline susceptibility in other diseases^24^,^25^. Therefore, we systematically evaluated HOXD9 targets for association with HGSOC risk using summary results from the meta-analysis (Methods). We identified 128 and 34 genes in IOE11-DNp53 and FT246-shp53-R24C, respectively, as cell-specific HOXD9 targets by applying a strict cutoff for differential expression (FDR<0.1, fold change >±2; *HOXD9* excluded). First, we compared the distribution of *P* values for association with HGSOC risk for SNPs in HOXD9 target genes and their flanking regions with the distribution in all other genes and their corresponding flanking regions using two-sample Kolmogorov–Smirnov (K–S) tests^26^. Flanking regions of 10, 25, 50 and 100 kb up- and downstream of each gene were tested under the assumption that HOXD9 binds to regulatory elements near its target genes. For all flanking intervals considered, SNP *P* values in and near HOXD9 targets were significantly smaller or more associated with HGSOC risk (K–S test *P* value: 4 × 10^−3^ to 3.9 × 10^−6^ for ovarian targets and 1 × 10^−3^ to 2.4 × 10^−7^ for fallopian targets; Table 3).

Next, we evaluated whether HOXD9 targets were enriched for HGSOC risk signals at three specific sub-genome-wide SNP *P* value thresholds of *P*<10^−3^, <10^−4^ and <10^−5^ compared with the proportion of such associations in all other genes. For all flanking regions as before, we observed significant enrichment for associations at the *P*<10^−3^ and <10^−4^ thresholds (Fisher’s exact *P* value range: ovarian targets: 6 × 10^−10^ to 1.2 × 10^−31^ and fallopian targets: 3.4 × 10^−9^ to 1.1 × 10^−21^; Table 3). At the *P*<10^−5^ threshold we only observed a significant enrichment for fallopian targets when flanking regions up to 100 kb were considered (*P*=5 × 10^−3^). Finally, we adopted a complementary approach and used gene set enrichment analysis (GSEA) to test the association of the ovarian and fallopian HOXD9 target gene sets (128 and 34 genes, respectively) with HGSOC risk. All genes in the genome with SNP coverage (22,577 genes) were first ranked based on the *P* value of the most significant HGSOC risk SNP in each gene and its flanking interval (±50 kb; Methods). On running GSEA with 10,000 permutations, the ovarian HOXD9 target gene set was significantly associated with HGSOC risk (GSEA *P*=0.017) but fallopian targets failed to reach significance (GSEA *P*=0.094). Thus, genes ranked higher in the GWAS meta-analysis were significantly over-represented among the 128 HOXD9 ovarian targets, in particular. All three approaches consistently demonstrated that HOXD9 target genes in ovarian cells were enriched for HGSOC risk variants.

Guided by the principle that disease genes are likely to cluster in functionally meaningful networks^27^, we also conducted network-based pathway analyses of all genes that showed at least twofold change in transcript abundance after *HOXD9* overexpression without considering the FDR threshold applied in the previous analyses (IOE11-DNp53: 2,357 genes; FT246-shp53-R24C: 1,972 genes, analysed separately). We assigned priority to genes in each downstream target list that are known to interact with each other biologically using jActiveModules^28^, a method that also takes into account the corresponding *P* values for differential expression after *HOXD9* perturbation. This identified a highly interconnected ovarian module or network of 94 genes and 272 interactions and a fallopian network of 269 genes and 962 interactions. Both the ovarian and fallopian networks identified were significantly enriched (FDR<0.05 and >5% pathway involvement) for the focal adhesion and transforming growth factor-beta signalling pathways from Kyoto Encyclopedia of Genes and Genomes (KEGG)^29^ and Ingenuity pathway databases (Table 4).

## Discussion

The main goals in the functional characterization of GWAS risk loci are to identify target susceptibility genes and the causal SNP(s) at risk loci. EQTL analysis represents one of the most straightforward approaches to the identification of the putative target genes at risk loci, and provides evidence of allele-specific functional effects for risk SNPs. We used data from HGSOCs from TCGA for eQTL analysis, and employed experimental models of early-stage disease to functionally validate the candidate genes we identified. Of 11 confirmed GWAS susceptibility loci identified for ovarian cancer, one contained a statistically significant eQTL association (*HOXD9*) at a FDR≤0.1. Two additional loci that were sub-genome-wide significant also contained significant eQTLs that coincided with risk SNPs (*CDC42* and *CDCA8*). For all three genes, at least one of the functional assays scored significantly, indicating they are the likely ovarian cancer susceptibility genes at these loci.

There may be several explanations why we did not identify eQTL associations at other loci. For example, we evaluated *cis*-eQTL associations for genes in a 500-kb region spanning the most significant risk SNP at each locus, since this threshold is expected to include the majority of eQTL associations^30^. However, it is known that enhancers can interact with multiple genes, and it is also plausible that risk-associated SNPs regulate genes many megabases away, or even on a different chromosome (that is, *trans*-eQTL associations). Also, this study was based on eQTL analysis in tumour tissues. Somatic genetic heterogeneity could mask the presence of eQTL associations; but it may also be that genes influence tumour development at early stages of neoplastic development requiring eQTL analysis to be performed in relevant normal tissues or putative precursor lesions. Moreover, eQTL analysis, unlike GWAS, is currently limited to sample sizes in the hundreds and the 339 HGSOCs used in this study, while comprising the largest available data set of its kind, may not be powered to detect all eQTL signals. Our approach was based on the hypothesis that risk variants function though cell-autonomous signalling pathways in differentiated cells, but it is possible that microenvironmental or precursor cell populations could also be effectors of risk variants, or that eQTLs can only be detected in the presence of certain stimuli, such as steroid hormones. Finally, our approach does not detect non-eQTL mechanisms underlying risk associations, such as splice variants and base changes in non-coding RNAs.

At two of the eQTL loci (1p34 and 2q31) the genes in closest proximity to the most risk-associated SNP were not the target gene from eQTL analysis. This has also been observed for other complex traits^8^. Furthermore the three candidate genes we identified have not previously been implicated in ovarian cancer susceptibility. At 2q31 susceptibility SNPs lie within the *HOXD* gene cluster, a series of conserved DNA-binding proteins involved in development. Homeobox genes have been broadly implicated in the development of many solid tumours, promoting neoplastic development by regulating processes common to normal tissue development and carcinogenesis, such as proliferation, invasion, differentiation and apoptotic resistance^31^. *HOXD9* lies ∼51 kb from the 19 risk-associated variants identified by fine mapping, which cluster around the *HOXD3* and *HAGLR* genes. This suggests that regulatory elements around *HOXD3/HAGLR* region regulate *HOXD9*. Using chromosome conformation capture (3C) assays we identified a putative interaction between one variant, rs2857532, and the *HOXD9* promoter, suggesting this SNP is a candidate causal variant regulating *HOXD9* expression at this locus. A recent study by Kelemen *et al.*^32^ reports that the 2q31.1 region is also a risk locus for the mucinous subtype of ovarian cancer with *HOXD9* the likely target susceptibility gene. Using 3C, Kelemen and colleagues also show that three regions, one of which harbours the rs2857532 risk SNP, interact with *HOXD9* in mucinous ovarian cancer cells indicating that there may be both tissue specific differences and similarities in the regulation of *HOXD9* in the two different disease subtypes. Rs2857532 lies within intronic sequence of *HOXD3,* but does not coincide with enhancer marks in normal ovarian or fallopian cells, or in serous ovarian cancer cells^33^. However, the risk allele of this SNP is predicted to create a binding site for two transcription factors implicated in early development: BEN, which is part of the TFII-I transcription factor family^34^, and HOMEZ, a putative, sequence-specific DNA-binding protein that may regulate the expression of HOX genes during vertebrate development^35^.

*HOXD9* is a little-studied homeobox gene known to be involved in the development of gynecological organs^36^ and mammary gland maturation during pregnancy and lactation^37^. Previous reports indicate *HOXD9* may behave as an oncogene in glioma^38^ and breast cancer^39^. Consistent with this, in functional assays we showed that higher HOXD9 expression reduced apoptosis, increased proliferation under conditions of high cell density and enhances ectopic proliferation of cells in the absence of attachment to a substrate. Analysis of downstream targets of HOXD9 identified by overexpressing this gene in ovarian and fallopian *in vitro* models and performing genome-wide RNAseq profiling indicated several candidate genes that may be necessary for HOXD9 to impart its neoplastic function. We tested these candidate genes for enrichment of HGSOC risk associations using a battery of complementary methods encouraged by the observation that the breast cancer susceptibility gene *FGFR2* has been shown to act through downstream transcriptional networks involving other breast cancer risk loci^24^. Notably, among the *HOXD9* ovarian targets enriched for modest (*P*<10^−4^) HGSOC risk variants were *WNT5A*, *SYNE1* and *IGF2*. *WNT5A* and *SYNE1* were also the top two genes driving the GSEA signal for the *HOXD9* ovarian gene set. *WNT5A*, a member of the non-canonical Wnt signalling pathway, has been shown to exhibit context-dependent tumour suppressor activity by triggering cellular senescence and is prognostic in primary HGSOC^40^,^41^. Smaller studies from OCAC have previously suggested associations between variants in *SYNE1* and *IGF2* with HGSOC risk but these have been significant only at sub-genome-wide levels^42^,^43^. The emergence of these two genes in the present analysis further underscores the utility of integrating functional data to highlight genetic risk associations and the likely existence of shared biological mechanisms underlying polygenic susceptibility. Pathway analysis revealed impact on focal adhesion signalling with involvement of the collagen genes *COL3A1* and *COL12A1* after *HOXD9* overexpression in both ovarian and fallopian cells. Focal adhesions play a critical role in ovarian cancer cellular migration and invasiveness^44^. Collectively, these findings further support the functional evidence indicating that *HOXD9* is the HGSOC susceptibility gene at the 2q31 locus.

At 1p36, we identified *CDCA8* as the target gene. *CDCA8* (alternatively known as Borealin) is part of the chromosomal passenger complex that functions to properly align and segregate chromosomes during mitosis. Consistent with this role, knockdown of *CDCA8* expression in IOE-DNp53 resulted in an accumulation of aneuploid cells in the culture. This is also consistent with the genomic instability and aneuploidy that is often observed in HGSOC, possibly arising from failure of chromosomal segregation during cell division. Finally, at 1p34, we identified *CDC42* as the putative target susceptibility gene. CDC42 is a small Rho GTPase and well-known oncogene involved in migration, cellular polarity and proliferation, and is overexpressed in many cancers^45^. Elevated expression of *CDC42* was associated with increased risk of HGSOC, and overexpression of the gene was associated with shorter population-doubling times and reduced migration.

Identifying additional common variant susceptibility alleles for ovarian cancer will continue to be restricted by sample size for this uncommon cancer type. By using eQTL analysis to interrogate candidate susceptibility loci that are sub-genome-wide significant, we have found evidence for two additional HGSOC risk loci, 1p34 (*CDCA8*) and 1p36 (*CDC42*) gene. While these functional studies were ongoing, a meta-analysis of the OCAC genetic association results with the results of an equivalent analysis of modifiers of ovarian cancer risk in 15,252 *BRCA1* mutation carriers and 8,211 *BRCA2* mutation carriers was conducted by the Consortium of Investigators of Modifiers of BRCA1/2 (ref. 19). This study identified six novel genome-wide significant risk loci for ovarian cancer, including the 1p34 and 1p36 loci described in the current study, thus validating our approach. In the meta-analysis, at 1p34 the most strongly associated SNP (rs58722170, 1.6 × 10^−8^ for all histological subtypes, 2.7 × 10^−12^ for serous) was correlated with the *cis*-eQTL SNP rs12023270 with *r*^2^=0.73; at 1p36 the most strongly associated SNP (rs56318008, 7.6 × 10^−9^ for all histological subtypes, 5.7 × 10^−8^ for serous) was correlated with the top *cis*-eQTL SNP rs2268177 with *r*^2^=0.76 (ref. 19).

In this study we evaluated the functional effects of candidate genes in ovarian and fallopian epithelial cells, because both cell types are predicted precursors of HGSOCs^46^,^47^. It is of interest that we observed some differences in how each cell type responded to altering the expression of the three candidate genes. For example, ovarian epithelial cells were more readily transformed in soft agar assays compared with fallopian cells even though the FT246-shp53-R24C cells express one additional oncogenic element compared with IOE11-DNp53 (mutant CDK4). HOXD9 target genes in ovarian cells were consistently more associated with HGSOC risk compared with fallopian HOXD9 targets. One possible explanation for these differences is that, even though in both cell lines p53 signalling was deregulated, the mechanism by which p53 was deregulated differs between the two models. An alternative explanation is that HGSOC originates in only one of these epithelial cell types and this is reflected by the different phenotypic effects observed when perturbing susceptibility genes. There remains debate about the cellular origins of HSGOC. The data in this study suggest that ovarian epithelial cells are more prone to neoplastic transformation by susceptibility genes associated with HGSOC compared with fallopian tube epithelial cells, and that ovarian cell transcriptional networks play a greater role in polygenic risk component of HGSOC. These variations in molecular and phenotypic changes between cell types highlights the need to consider carefully the likely cell of origin for the disease under study when performing functional studies of risk loci identified by GWAS. Moreover, the heterogeneity in the phenotypic effects observed for the different genes reveal the importance of evaluating multiple phenotypes associated with neoplasia, as risk alleles could influence cellular transformation through a variety of mechanisms.

In summary, this study has demonstrated the power of eQTL analysis to identify candidate susceptibility genes associated with initiation and early stage development of HGSOC. In particular we show how biological information from the functional characterization of risk loci can be used to interrogate sub-genome-wide significant loci from GWAS for the identification of additional, novel risk loci for common multifactorial disease traits.

## Methods

### Genetic association analyses

*Summary of data sets*. Data were available for the stage 1 of three population-based EOC GWAS comprising a total of 4,366 cases and 9,124 controls^9^,^18^,^19^. An additional 11,030 cases and 21,693 controls from 41 OCAC studies were genotyped using the iCOGS array. All duplicates were removed from the analysis and overall, 43 studies from 11 countries provided data on 15,397 women of European ancestry, diagnosed with invasive epithelial EOC, 9,608 of whom were diagnosed with serous EOC and 30,816 controls from the general population. The quality control methods are described in full in the Supplementary File 1.

*Imputation*. We performed imputation separately for OCAC–iCOGS samples and each of the GWAS. We imputed variants from the 1000 Genomes Project data using the v3 April 2012 release as the reference panel. To improve computation efficiency we initially used a two-step procedure, which involved pre-phasing in the first step and imputation of the phased data in the second. We carried out pre-phasing using the SHAPEIT software^48^. We then used the IMPUTE version 2 software^49^ for the subsequent imputation for all studies. To perform the imputation we divided the data into segments of ∼5 Mb each. We excluded SNPs from the association analysis if their imputation accuracy was *r*^2^<0.25 or their minor allele frequency was <0.005. The number of successfully imputed SNPs by minor allele frequency is shown in Supplementary File 1.

*Data analysis*. All analyses were restricted to subject of European intercontinental ancestry. To be able to control for population substructure we used a set of unlinked markers to perform principal components analysis. The three GWAS and the COGS data sets were analysed separately using different sets of markers. To enable this analysis on very large samples we used an in-house programme written in C++ using the Intel MKL libraries for eigenvectors (available at http://ccge.medschl.cam.ac.uk/software/). Unconditional logistic regression treating the number of alternate alleles carried as an ordinal variable (log-additive, co-dominant model) was used to evaluate the association between each SNP and ovarian cancer risk. A likelihood ratio test was used to test for association, and per-allele log odds ratios and 95% confidence limits were estimated. The likelihood ratio test has been shown to have greater power than alternatives such as the Wald test and score test for rare variants^50^. The logistic regression model was adjusted for study and population substructure by including study-specific indicators and a variable number of eigenvalues from the principal components analyses. The number of principal components was chosen based on the position of the inflexion of the principal components scree plot. Two principal components were included in the analysis of the UK and US GWAS data sets, one was used for the Mayo GWAS and five were used for the COGS–OCAC data set. Results from the three GWAS and COGS were combined using fixed-effect inverse variance weighted meta-analysis.

### eQTL analysis

We chose 47 candidate HGSOC risk loci from previous GWAS studies with *P* value <1 × 10^−5^ (Supplementary Table 1). For each risk SNP, correlated variants with *R*^2^>0.7 in the 1000 Genomes CEU population were identified. The germline genotypes of 443 ovarian serous cystadenocarcinoma samples were downloaded from TCGA data portal. We selected 339 samples with Caucasian ancestry using EIGENSTRAT^51^. Matched tumour gene expression profiles, somatic copy number and CpG methylation data of these samples were obtained from the same source and used to adjust the expression profiles for somatic copy number changes and CpG methylation variation described as follows^15^,^16^. Briefly we adjusted the expression levels for each gene using matched information of somatic copy number and CpG methylation using linear models. To perform the eQTL analysis, we took germline genotypes of SNPs/proxies as independent variables and adjusted expression levels as traits. The association between genotype and gene expression of genes within 250 kb either side of the corresponding variant was evaluated based on the significance of linear regression coefficients. To control for multiple testing, we calculated the FDR from the test *P* values using Benjamini–Hochberg method and called significant associations with a maximal FDR of 0.1.

### Cell lines and cell culture

We have previously reported the generation of the IOE11 TERT-immortalized ovarian surface epithelial cell line^23^. IOE11 cultured in NOSE-CM^52^. To generate a p53-deficient line, IOE11 cells were transfected with T7-p53DD-pcDNA3 (Addgene plasmid number 25989) and positive clones (IOE11-DNp53) selected with 125 μg ml^−1^ G418. Loss of p53 function was confirmed by irradiating IOE11-DNp53 and control cells with 6 Gy ionizing radiation and immunoblotting cell lysates for p21 expression (sc-397, 1:1,000 dilution, Santa Cruz Biotechnology) 24 h later. Immortalized fallopian tube secretory epithelial cell lines (FT33-shp53-R24C and FT246-shp53-R24C) have been previously described^22^ and were cultured in DMEM/F12 (Sigma) supplemented with 2% Ultroser G (Crescent Chemicals) or 10% fetal bovine serum (FBS; Hyclone, Thermo Fisher). For 3C, HEY cells were grown in RPMI containing 10% FBS and OVCA429 cells were cultured in EMEM supplemented with 10% FBS, 1 × non-essential amino acids and 1 × sodium pyruvate. All cell lines used in this study were routinely tested for *Mycoplasma* infection using a *Mycoplasma*-specific PCR, and, for cell line authentication, short tandem repeats profiled using the PowerPlex16HS Assay (Promega, University of Arizona Genetics Core).

### Viral transductions

A set of six *CDCA8*-targeting shRNAs and one scrambled shRNA (SCR) cloned into pGIPz (RHS4531-EG55143, Dharmacon) were co-transfected with p8.91 and pMD.G into HEK293Ts to produce lentiviral supernatants, which were collected 48 h after removal of the transfection media. Lentiviral GFP fusion constructs were purchased from Genecopoeia: Lv122-CDC42-GFP and Lv122-HOXD9-GFP (and a GFP control; Lv-GFP) and also used to make lentiviral supernatants. IOE11-DNp53 and FT246-shp53-R24C cells were transduced with lentiviral supernatants overnight, and for IOE11-DNp53, positive cells were selected using 400 ng ml^−1^ puromycin.

### Functional assays

For anchorage-dependent growth assays, 0.1 × 10^6^ cells were plated in triplicate and passaged when 80% confluent. Cells were enumerated at each passage and population doublings calculated with the following formula: population doubling=log (total cell number at each passage/initial cell number)/log2. Anchorage-independent growth assays were performed by suspending 0.02 × 10^6^ cells in media containing 0.33% Noble agar and 1 mg ml^−1^ bacto-peptone (both Sigma); this mixture was overlayed onto a base layer of medium containing 0.6% Noble agar per 1 mg ml^−1^ bacto-petone. Cells were cultures for 4 weeks, stained with 1% *p*-iodonitrotetrazolium violet (Sigma) and counted using phase microscopy. Migration and invasion kits (Trevigen) were performed following the manufacturer’s instructions. Contact inhibition assays were performed by plating 0.02 × 10^6^ cells per well in 12-well plates and enumerating cells at indicated timepoints. For propidium iodide staining: 0.3 × 10^6^ cells were plated in triplicate and incubated for 48 h. Cells were washed twice with PBS and fixed in 70% ice-cold ethanol. On fixation cells were washed twice with PBS and stained with 50 μg ml^−1^ propidium iodide staining solution (Calbiochem) combined with 10 μg ml^−1^ RNase A (Invitrogen). Cells were stained for 3 h at 4 °C in the dark. Cell cycle status was examined using the LSR II flow cytometer (Becton Dickinson) and data were analysed using FlowJo software (Tree Star, Inc.).

### Chromosome conformation capture (3C)

3C was performed as follows^7^. Briefly, HEY and OVCA429 EOC cells were collected by trypsinisation, and 10 million cells were fixed with 1% formaldehyde for 10 min. Cells were lysed (10 mM Tris-HCl (pH 8), 10 mM NaCl and 0.2% Nonidet P-40) to release the nuclei, and pelleted nuclei were resuspended in restriction enzyme buffer containing 0.1% SDS and 1.6% Triton-X. A total of 1,500 units of Csp6i (Fisher BioReagents) were added and incubated at 37 °C for overnight. Digestions were halted by incubation with 1.5% SDS at 65 °C for 30 min. Digested samples were added to the ligation buffer containing 4000U T4 DNA ligase (NEB) and 1% Triton X-100 to neutralize SDS, and incubated for 24 h at 16 °C. Samples were decrosslinked by overnight incubation at 65 °C with proteinase K. Libraries were extracted using standard phenol/chloroform protocols, precipitated using ethanol, and desalted using Microcon Ultra Cell YM-100 columns. Primers were designed at the HOXD9 promoter and for each restriction fragment containing risk-associated SNPs (Supplementary Table 2). PCR was performed using Taq polymerase (QIAGEN), using the following conditions: 5 min at 94 °C, 35 cycles of (20 s at 94 °C, 20 s at 61 °C and 30 s at 72 °C), and 10 min at 72 °C. The PCR products were run on a 1.7% agarose gel, gel purified using the QIAgen Gel Extraction kit, and sequenced.

For analysing long-range interaction quantitatively a BAC library (RP11-892F14, CHORI) was prepared as follows: briefly, BAC DNA was purified from a 500 ml *Escherichia coli* culture and 20 μg of BAC DNA was then digested with Csp6i overnight at 37 °C followed by ligation with T4 DNA ligase overnight at 16 °C (refs 53, 54). 3C libraries as well as the BAC library were titrated by serial dilution to identify the concentration of template for quantitative PCR analysis for each genomic region of interest. The PCR products were run on an agarose gel and stained with ethidium bromide. Intensity measurements for each of the bands were quantified using ImageQuant LAS4000 (Roche) with Image QuantTL8.1 software (Roche). The interaction frequency was determined by dividing the amount of PCR product obtained using the 3C template by the amount of PCR product obtained using the control template. Data were normalized using the lowest interaction value amongst the 11 amplicons (that is, the lowest interaction was set to 1). Each template was run in triplicate and the standard error of measurement (s.e.m.) calculated. The s.e.m. for each amplicon was <15%.

### Transcription factor-binding site analysis

Transcription factor-binding site analyses were performed in Biobase, using the TRANSFAC Match tool. Two 21-bp sequences, representing the two alleles of rs2857532±10 bp, were uploaded. The TRANSFAC MATRIX TABLE library was used (Release 2014.2), with the vertebrate_non_redundant.prf profile and cutoffs selected to minimize the sum of both error rates (false positive and false negatives).

### RNAseq analysis in HOXD9 models

One million cells were plated into a P100 dish and cultured for 48 h. Cells were washed twice with ice-cold PBS and lysed *in situ.* RNA extractions were performed using the QIAgen miRNAeasy kit with on-column DNase I digests, following the manufacturer’s instructions. RNA sequencing was performed by BGI Americas. Briefly, 3 μg of RNA was depleted of ribosomal RNA and libraries created using the Illumina TruSeq kit. Sequencing was performed by multiplexing six samples per lane for sequencing on an Illumina HiSeq2000. Linear fold change in transcript abundance before and after HOXD9 overexpression and *P* values from analysis of variance for differential gene expression were calculated using the workflow implemented in the Partek Genomics Suite.

### Enrichment analysis

Enrichment analysis was restricted to genes that demonstrated at least twofold change in transcript abundance and showed significant differential expression (FDR<0.1) after *HOXD9* overexpression (IOE11-DNp53: 128 genes; FT246-shp53-R24C: 34 genes). Ovarian and fallopian gene lists were analysed separately. First, all SNPs (*n*=9,772,651) with minor allele frequency>0.01 from the HGSOC risk meta-analysis described above were mapped to genes from the UCSC hg19 knownGene track. SNPs were assigned to genes if they were in the gene or 50 kb on either side of it. We then compared the distribution of *P* values for association with HGSOC risk for SNPs in HOXD9 target genes and their flanking regions with the distribution in all other genes and their corresponding flanking regions using two-sample K–S tests^26^. The analysis was repeated using extended boundaries of 10, 25 and 100 kb on either side of each gene. Second, proportions of SNPs associated with HGSOC risk at *P* value thresholds of *P*<10^−3^, <10^−4^ and <10^−5^ in HOXD9 target genes was compared with the corresponding proportions in all remaining genes using two-tailed Fisher’s exact tests for each of the flanking boundaries considered in the first analysis. Third, we ranked all genes in descending order of the −log_10_ of the *P* value of the most significant SNP in each gene (±50 kb). A total of 22,577 genes were covered by SNPs with the 50-kb flanking regions considered. Gene set enrichment analysis with 10,000 permutations was used to test enrichment of genes ranked highly in this list among the ovarian and fallopian tube HOXD9 targets^55^.

### Pathway analysis

Pathway analysis involved genes that demonstrated at least twofold change in transcript abundance after *HOXD9* overexpression (IOE11-DNp53: 2,357 genes; FT246-shp53-R24C: 1,972 genes). These genes and corresponding *P* values for differential expression were used as input for the jActiveModules^28^ (v 2.2.3) plugin in Cytoscape^56^ (v 3.1.0). Ovarian and fallopian gene lists were analysed separately. The jActiveModules approach combines input *P* values with prior knowledge of biological interactions between input genes to identify modules or networks of input genes with high functional connectivity and significant differential expression. We set up the plugin to identify the single best network using default parameters (except regional scoring). Known biological interactions in the data were prioritized using 290,438 non-redundant binary interactions between 17,977 genes/proteins compiled from up-to-date, high-quality, curated resources that combine comprehensive genetic, molecular, protein–protein and protein–DNA interaction annotation. These were Multinet^57^, InWeb^58^, HINT^59^ and 252 KEGG^60^ pathways converted to binary format using the Bioconductor package graphite^61^. Pathways from the Ingenuity Knowledge Base and KEGG significantly enriched in the single best network discovered by jActiveModules for the ovarian and fallopian gene lists were identified using a right-tailed Fisher’s exact test with FDR control for multiple pathway comparisons by the Benjamini–Hochberg method. The KEGG-based analysis was conducted using the Database for Annotation, Visualization and Integrated Discovery (v 6.7) (ref. 62). We reported pathways common to both the ovarian and fallopian HOXD9 networks that were significant at FDR<0.05 with >5% of the pathway involved.

## Additional information

**How to cite this article:** Lawrenson, K. *et al.* *Cis*-eQTL analysis and functional validation of candidate susceptibility genes for high-grade serous ovarian cancer. *Nat. Commun.* 6:8234 doi: 10.1038/ncomms9234 (2015).

## Supplementary Material

Supplementary Information Supplementary Figures 1-2, Supplementary Tables 1-5, Supplementary Note 1, Supplementary Methods and Supplementary References.

## Figures and Tables

**Table 1:** Risk and eQTL associations in serous ovarian cancer.

**Locus**	**Risk associations**				**eQTL associations**					***r*** ^**2**^
	**rsID**	**OR**	***P*** **value (1 df)**	**EAF**	**rsID**	**Gene**	**Stat**	**Unadjusted** ***P*** **value**	**FDR**	
2q31	rs6755777	1.15	8.95 × 10^−14^	0.68	rs711830	*HOXD9*	3.48	5.82 × 10^−4^	0.03	0.99
1p36	rs72665317	0.89	6.83 × 10^−7^	0.16	rs2268177	*CDC42*	−7.46	8.40 × 10^−13^	9.07 × 10^−11^	0.88
					rs7412010	*CDC42*	7.38	1.36 × 10^−12^	9.07 × 10^−11^	0.78
1p34	rs4335340	0.90	1.37 × 10^−7^	0.25	rs12023270	*CDCA8*	3.22	1.41 × 10^−3^	0.05	0.61
20q13	rs6026494	1.16	5.07 × 10^−7^	0.11	rs6026494	*GNAS*	2.96	3.28 × 10^−3^	0.09	1.00

EAF, effect allele frequency; OR, odds ratio; Stat, *T*-statistic.

*r*^2^ values between risk SNP and eQTL SNP are from 1000 Genomes Phase 1 EUR population. Risk associations from an OCAC-only analysis.

**Table 2:** TRANSFAC analysis of predicted allele-specific transcription factor binding at rs2857532.

**Matrix**	**Factor name**	**Strand**	**Core score**	**Matrix score**	**Sequence**
V$HOMEZ_01	Homez	(−)	0.888	0.674	aacaggAGC**G**Aaattcc
V$BEN_01	BEN	(+)	0.877	0.878	GAGC**G**aaa
V$RELA_Q6	RelA-p65	(−)	1	0.928	agc**g**aaATTCCa

Analyses were performed using the Match tool. Only transcription factors (TFs) predicted to uniquely bind to the risk (G) allele are shown. The position of the polymorphism within the TF-binding sequence is shown in bold font.

**Table 3:** Enrichment of HGSOC risk variants in regulatory regions of HOXD9 target genes.

**Cell type**		Ovarian HOXD9 target genes* **(*****n*****=128)**				Fallopian HOXD9 target genes* **(*****n*****=34)**			
**Gene ±extended boundaries**		**10 kb**	**25 kb**	**50 kb**	**100 kb**	**10 kb**	**25 kb**	**50 kb**	**100 kb**
K–S test		4.2 × 10^−4^	0.004	0.006	3.9 × 10^−6^	0.001	3.7 × 10^−6^	2.4 × 10^−7^	5 × 10^−6^
Fisher’s exact test threshold	*P*<10^−3^	4.9 × 10^−10^	7.9 × 10^−14^	1.9 × 10^−18^	5.2 × 10^−14^	1.1 × 10^−15^	3 × 10^−11^	4.4 × 10^−13^	3.4 × 10^−9^
	*P*<10^−4^	6 × 10^−10^	8.5 × 10^−14^	1.2 × 10^−20^	1.2 × 10^−31^	5 × 10^−20^	8.5 × 10^−17^	1.1 × 10^−21^	3.8 × 10^−16^
	*P*<10^−5^	No SNPs	No SNPs	No SNPs	No SNPs	0.876	0.779	0.178	0.005

^*^FDR<0.1 for differential expression and fold change >±2 after HOXD9 overexpression.

**Table 4:** Pathway analysis of HOXD9 target gene networks.

**Source**	**Pathway** *	**Ovarian HOXD9 network**		**Fallopian HOXD9 network**	
		**% of pathway involved**	**FDR**	**% of pathway involved**	**FDR**
*KEGG*					
	Focal adhesion	14	1.9 × 10^−4^	9	1.9 × 10^−7^
	TGF-beta signalling pathway	9	2.3 × 10^−3^	11	5.1 × 10^−3^
*Ingenuity*					
	FAK signalling	5	6.9 × 10^−3^	12	6.5 × 10^−6^
	ERK5 signalling	5	1.8 × 10^−2^	13	3 × 10^−5^
	RAR activation	5	1.9 × 10^−4^	7	6 × 10^−5^
	TGF-beta signalling	7	2.4 × 10^−4^	9	2.1 × 10^−4^
	Hepatic fibrosis/hepatic stellate cell activation	8	4 × 10^−12^	6	5.5 × 10^−4^
	Cell cycle: G1/S checkpoint regulation	8	4.6 × 10^−4^	9	1.1 × 10^−3^
	Chronic myeloid leukaemia signalling	6	2.7 × 10^−4^	8	1.3 × 10^−3^
	Pancreatic adenocarcinoma signalling	5	2.4 × 10^−3^	7	2.5 × 10^−3^
	Virus entry via endocytic pathways	6	1.2 × 10^−3^	7	4.3 × 10^−3^
	Growth hormone signalling	6	3.5 × 10^−3^	7	6.6 × 10^−3^
	Caveolar-mediated endocytosis signalling	7	6 × 10^−4^	7	7.7 × 10^−3^
	Cyclins and cell cycle regulation	6	7.2 × 10^−4^	6	1.1 × 10^−2^
	Antiproliferative role of TOB in T-cell signalling	12	3 × 10^−3^	12	1.2 × 10^−2^
	Semaphorin signalling in neurons	6	1.2 × 10^−2^	8	1.3 × 10^−2^
	Remodelling of epithelial adherens junctions	6	3.4 × 10^−3^	6	2.6 × 10^−2^
	VDR/RXR activation	5	5.1 × 10^−3^	5	3.9 × 10^−2^

TGF, transforming growth factor.

^*^Only pathways with FDR<0.05 and >5% genes involved in both ovarian and fallopian analysis reported.
