# Supplementary material for: Cis-eQTL analysis and functional validation of candidate susceptibility genes for high-grade serous ovarian cancer
Source: Nat Commun. 2015 Sep 22;6:8234. doi: 10.1038/ncomms9234 (PMC4580986; doi:10.1038/ncomms9234)
Supplement: Supplementary Information — Supplementary Figures 1-2, Supplementary Tables 1-5, Supplementary Note 1, Supplementary Methods and Supplementary References. [file ncomms9234-s1.pdf]

Supplementary Figure 1. Establishment of a p53-deficient ovarian epithelial cell line. (a) IOE11 cells were transfected with a dominant negative p53 (DNp53) vector (pcDNA3.1-DNp53) or the vector backbone as a control (pcDNA3.1). (a) Detection of endogenous and DNp53 by Western blotting. MCF10A cell lysate was used as a positive control for wild type p53. (b) Parental IOE11 and IOSE11-DNp53 cells were treated with 6 Gy ionising radiation (IR). Parental cells upregulate p21 expression following IR treatment, whereas IOE11-DNp53 cells do not, demonstrating a loss of functional p53 signaling following DNA damage. (c) Growth curves. Cells expressing DNp53 have a shorter population doubling time than control cells. (d) Cell cycle analysis using propidium iodide staining. We detected a small, albeit non-statistically significant, increase in the proportion of cells in G2 following expression of the DNp53 construct. (e) After passage 13, we detected a significantly increased proportion of aneuploid cells (>4N) in the IOE11-DNp53 cultures compared to controls (two-tailed paired T-test). Error bars show mean ± standard deviation.

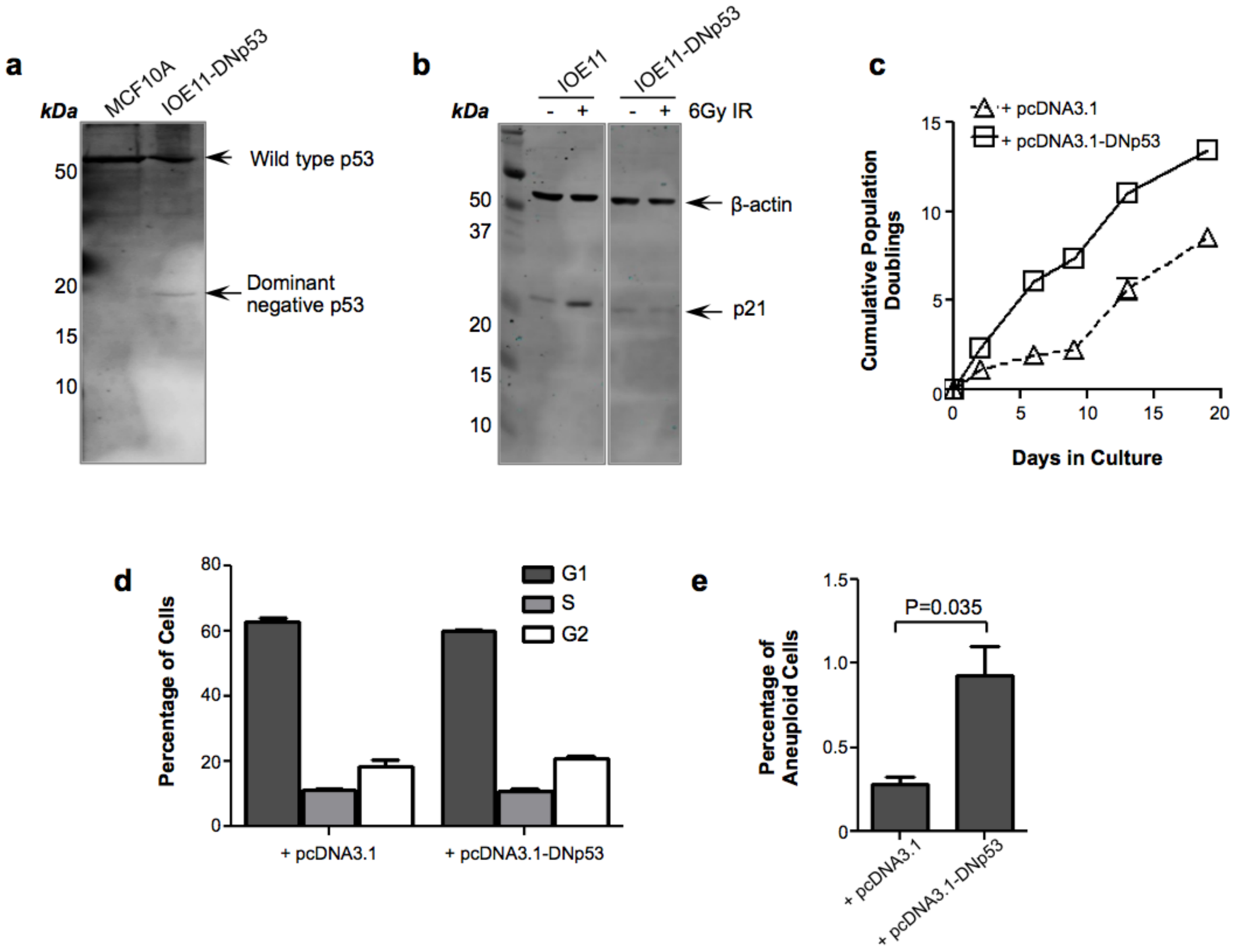

Supplementary Figure 2. Validation of *HOXD9* genes identified by RNAseq. (a) IOSE11-DNp53 and (b) FT246 models. The ratio of gene expression in HOXD9 OE cells compared to GFP expressing cells is shown, measured by RNAseq and RT-qPCR, performed using TaqMan gene expression probes.

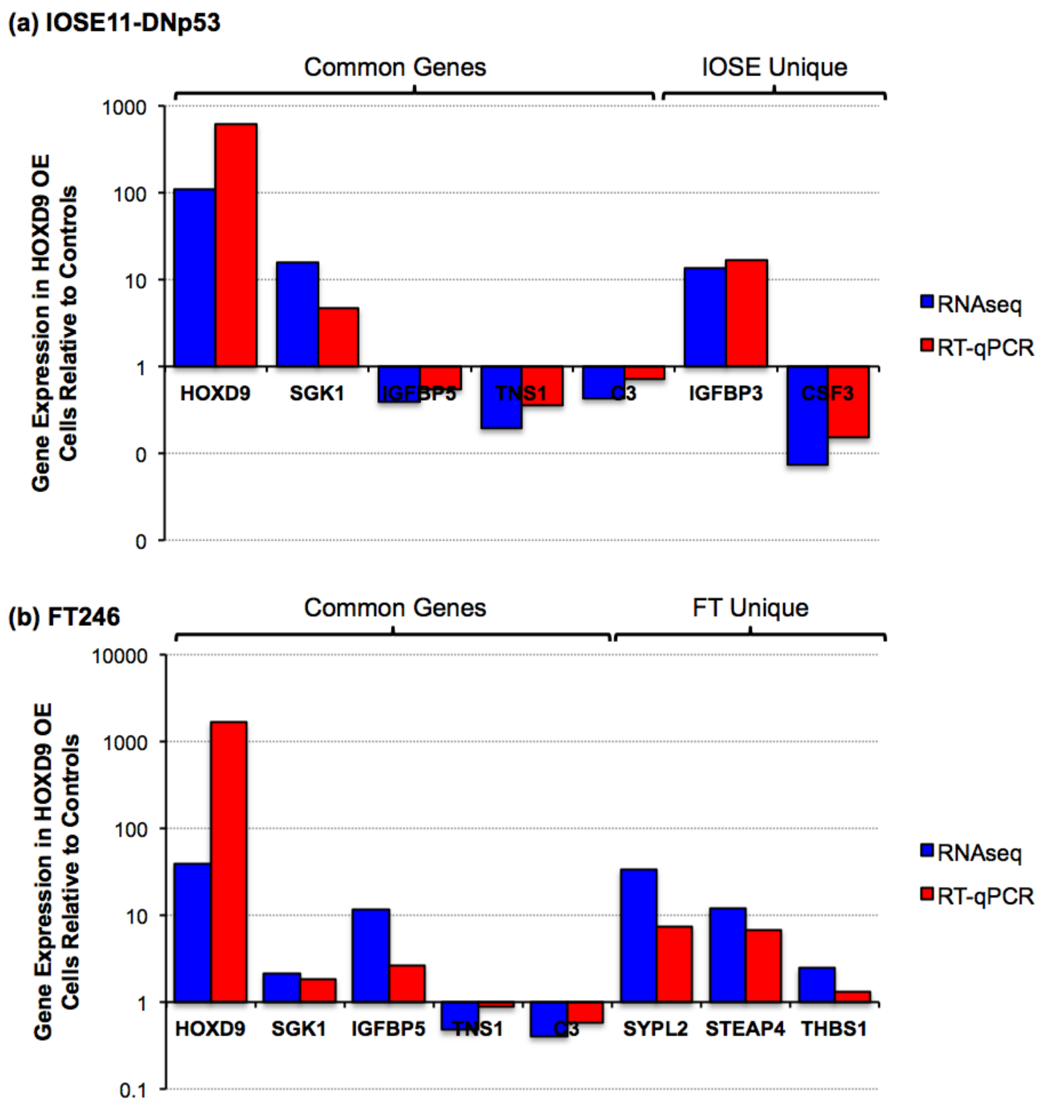

Supplementary Table 1. The forty-seven HGSOC risk SNPs used in the eQTL analyses.

SNP	Chromosome	Position
rs2268177	1	22415410
rs4335340	1	38098035
rs140656631	1	152076102
rs147001887	1	233131754
rs6755777	2	177043226
rs10172595	2	232387063
rs113156482	3	27385515
rs149825468	3	79402822
rs62274042	3	156435952
rs17329882	4	119949960
chr4:185470586:I	4	185470586
rs10069690	5	1279790
rs463548	5	54491914
rs74959519	5	84618055
chr5:164862654:I	5	164862654
rs143589614	5	175418049
rs80077411	6	595273
rs115344852	6	28486098
rs187759744	6	32539152
rs7763414	6	151405377
rs62430802	7	929439
rs1881116	7	1053958
rs62451762	7	7108188
rs76837345	8	82668818
rs10088218	8	129543949
rs4631563	9	16913286
chr9:136138765:D	9	136138765
rs1802669	10	21827796
rs16937956	11	8404501
rs11024093	11	16992395
rs10501153	11	36386755
chr11:86642872:I	11	86642872
rs9559508	13	109805793
rs1315145	14	33846320
chr14:42173641:I	14	42173641
rs71402493	16	10620039
rs4785001	16	58964191
rs75846557	16	62734617
rs4782985	16	84537527
chr17:29181220:I	17	29181220
rs757210	17	36096515
rs146746174	17	43569909
rs7207826	17	46500673
rs78013533	17	60480968
rs4808075	19	17390291
rs6026492	20	57330569
rs6005807	22	28934313

Supplementary Table 2. Primers used for 3C.

For mapping interactions using agarose gel electrophoresis

HOXD9-1	TGAGCTGGGCAACTTGTAGA
Target-1	CACCTCCAAAGAAATGGGTCT
Target-2	GTGGGAAAGGGAGAAAGGAT
Target-3	CCCACCAATACATCAATACCG
Target-4	ACTGGCCAGAGCTGAGAGTC
Target-5	ACTGGGTGGCGTGAAAGTT
Target-6	GGACCAGCTGGCTTGAGA
Target-7	TTTTCAGGAAGATGCCCAAT
Target-8	TGCAGGTGGAGGGAAAACTA
Target-9	AGTTTAGTGGGCGAATCAGC
Target-10	TCCCCAAGCCTAGGTGATTA
Target-11	GCCAAATCACAGCCCAATA
Target-12	CTGCCTGCCACAACAGAAG
Target-13	CCACAGTCCTCACCACAGTTT
Target-14	GAGGTTTGCACACACAGTCG
Target-15	ATCTTGGGCAAAATGTCCTG
Target-16	TTTGATGGCTCCATTGTTCA
Target-17	GAGGTGTGGTCAAAATGTTCC
Target-18	TTCAGAGCCCTCTCCTTCAA
Target-19	AGCATGGTGCGAAAAAGTCT

For quantitative 3C:

Primer #	Sequence	Genomic distance kb from HOXD9 promoter (kb)
1	GAAGGTCATTGTAAGTGAGGGTTT	4
2	GAGGTCTATTTCCAAGGCTGTG	7
3	TACGGATACGATAACTTACAGAGACAG	10
4	CCTCCACTCTTGGGCTTCTA	32
5	GAGAGGAGGAGGGAGAGGAA	41
6	AGTTTAGTGGGCGAATCAGC	46
7	TCCCCAAGCCTAGGTGATTA	48
8	CTGCCTGCCACAACAGAAG	50
9	GAGGTTTGCACACACAGTCG	53
10	AGCATGGTGCGAAAAAGTCT	60
11	CCTCTAACGACCTGACACTTTTTC	85

Supplementary Table 3. Studies contributing to the three EOC GWAS and COGS

OCAC Study Name	Abbrev.	Study Location	Study Type	Controls	Cases[1]
Australian Ovarian Cancer Study/Australian Cancer Study (Ovarian Cancer)	AUS	Australia	Population based/ case-control	977	880 (880)
Bavarian Ovarian Cancer Cases and Controls	BAV	Southeast Germany	Population based/ case-control	143	93 (93)
Belgian Ovarian Cancer Study	BEL	Belgium, University Hospital Leuven	Hospital based/ Case-control	1,349	275 (274)
Diseases of the Ovary and their Evaluation	DOV	USA: 13 counties in western Washington state	Population based/ case-control	1,487	904 (900)
Oregon Ovarian Cancer Registry	ORE	USA: Portland, Oregon	Case only	0	55 (52)
German Ovarian Cancer Study	GER	Germany: two geographical regions in the states of Baden-Württemberg and Rhineland-Palatinate in southern Germany	Population based/ case-control	413	189 (183)
Dr. Horst Schmidt Kliniken	HSK	Germany	Case only		144 (144)
Hawaii Ovarian Cancer Case-Control Study	HAW	USA: Hawaii	Population based/ case-control	157	60 (60)
Hannover-Jena Ovarian Cancer Study	HJO	Germany	Hospital based/ Case-control	273	266 (0)
Hannover-Minsk Ovarian Cancer Study	HMO	Belarus	Case-control	138	143 (0)
Helsinki Ovarian Cancer Study	HOC	Helsinki, Finland	Case-control	447	221 (0)
Hormones and Ovarian Cancer Prediction	HOP	USA: Western PA, Northeastern Ohio, Western New York	Population based/ Case-control	1,464	654 (633)
Gilda Radner Familial Ovarian Cancer Registry	GRR	USA: National	Familial cancer/ Case only		132 (0)
Women's Cancer Program at the Samuel Oschin Comprehensive Cancer Institute	LAX	USA: Southern California	Case only		277 (276)
Los Angeles County Case-control studies of Ovarian Cancer-1	USC	USA: Los Angeles County, California	Population based/ Case-control	1047	689 (677)
MALignant OVArian Cancer	MAL	Denmark	Population based/ Case-control	828	440 (440)
Danish Pelvic Mass Study	PVD	Denmark	Case only	0	169 (0)
Mayo Clinic Ovarian Cancer Case-Control Study	MAY	USA: North Central (MN, SD, ND, IL, IA, WI)	Clinic based/ Case-control	971	811 (808)
Melbourne Collaborative Cohort	MCC	Melbourne, Australia	Cohort/ Nested	66	63

OCAC Study Name	Abbrev.	Study Location	Study Type	Controls	Cases[1]
Study			case-control		(0)
MD Anderson Ovarian Cancer Study	MDA	USA: Texas	Hospital based/ Case-control	384	373 (0)
Memorial Sloan-Kettering Cancer Center	MSK	USA: New York City	Case-control	593	467 (401)
North Carolina Ovarian Cancer Study	NCO	USA: Central and eastern North Carolina (48 counties)	Population based/ Case-control	826	761 (756)
New England Case Control Study	NEC	USA: New Hampshire and Eastern Massachusetts	Population based/ Case-control	1,122	766 (677)
Nurses' Health Study	NHS	USA: National	Population based/ Nested case-control	455	138 (0)
New Jersey Ovarian Cancer Study	NJO	USA: New Jersey (six counties)	Case-control	181	169 (158)
University of Bergen, Haukeland University Hospital, Norway	NOR	Norway	Case-control	371	237 (0)
Nijmegen Ovarian Cancer Study	NTH	Eastern part of the Netherlands	Case-control	323	255 (252)
Ovarian Cancer in Alberta and British Columbia	OVA	Alberta and British Columbia, Canada	Case-control	748	631 (0)
Polish Ovarian Cancer Study	POC	Poland: Szczecin, Poznan, Opole, Rzeszów	Case-control	417	422 (401)
Polish Ovarian Cancer Case Control Study	POL	Poland: Warsaw, and Lodz	Population based/ Case-control	741	260 (258)
Study of Epidemiology and Risk Factors in Cancer Heredity	SEA	UK: East Anglia and West Midlands	Population based/ Case-control	6,024	1,385 (1,079)
Family Registry for Ovarian Cancer and Genetic Epidemiology of Ovarian Cancer	STA	USA: Six counties in the San Francisco Bay area	Population based/ Case-control	313	251 (250)
Toronto Ovarian Cancer Study	TOR	Canada: Province of Ontario	Population based	598	755 (0)
University of California Irvine Ovarian Study	UCI	USA: Southern California (Orange and San-Diego, Imperial Counties)	Population based/ Case-control	367	277 (276)
United Kingdom Ovarian Cancer Population Study	UKO	UK: England, Wales and Northern Ireland	Population based/ Case-control	1,104	730 (712)
Royal Marsden Hospital Ovarian Cancer Study	RMH	UK: London	Hospital based/ Case only	0	154 (138)
UK Familial Ovarian Cancer Registry	UKR	UK: National	Case only/ Familial register	0	47 (45)
Southampton Ovarian Cancer Study	SOC	UK: Wessex region	Case only/ Hospital based	0	267 (76)

OCAC Study Name	Abbrev.	Study Location	Study Type	Controls	Cases[1]
Scottish Randomised Trial in Ovarian Cancer	SRO	UK: coordinated through clinical trials unit, Glasgow UK from patients recruited world-wide	Case only/ Clinical trial	0	158<br>(135)
Warsaw Ovarian Cancer Study	WOC	Poland: Warsaw and central Poland	Case-control	204	202<br>(151)
Welcome Trust Case Control Consortium	WTC	United Kingdom	Blood donors and 1958 Birth Cohort Controls	6,118	
**Total**				**30,816**	**15,397 (11,398)**

1. Number cases with survival time in parentheses

Supplementary Table 4. Summary of genotyping data for each data set

GWAS	Sub-study	Number (European ancestry)		Genotyping array	Genotyping centre	Number SNPs passing QC
		Cases	Controls			
OCAC-iCOGS	Multiple	12,618 * (11,030)	24,319 * (21,693)	Illumina custom iSelect, "iCOGS"	McGill University and Génome Québec Innovation Centre and Mayo Clinic Medical Genome Facility	199,570
UK	RMH	147		Illumina 610K	Illumina Corporation	507,094
	SEA	1,078				
	UKO	494				
	UKR	44				
	WTC		6,118	Illumina 550K	Sanger Centre	507,094
	Total	1,763	6,118			
Mayo		441	442	Human Omni 2.5-8 BeadChip	Mayo Clinic Medical Genome Facility	1587042
US	MAY	359	519	Illumina 610K	Mayo Clinic Medical Genome Facility	556,480
	NCO	492	654			
	TBO	227	169			
	TOR	734	524			
	NEC/BWH	132	142	Illumina 317K	National Cancer Insitute	305,690
	POL	218	555	Illumina 550K		527,435
	Total	2,162	2,564			

* After exclusion of samples also genotyped in other GWAS

Supplementary Table 5: Number of imputed variants by minor allele frequency

Minor allele frequency in controls	Number
<1 per cent	5779085
[1 – 5) per cent	2954998
[5 – 10) per cent	1396009
[10 - 50] per cent	5374631

Supplementary Note 1

Acknowledgements

Funding of constituent studies

These are listed by funding agency, with each grant number in parentheses:

Funding of the constituent studies was provided by the American Cancer Society (CRTG-00-196-01-CCE); the California Cancer Research Program (00-01389V-20170, N01-CN25403, 2II0200); the Canadian Institutes for Health Research (MOP-86727); Cancer Council Victoria; Cancer Council Queensland; Cancer Council New South Wales; Cancer Council South Australia; Cancer Council Tasmania; Cancer Foundation of Western Australia; the Cancer Institute of New Jersey; Cancer Research UK (C490/A6187, C490/A10119, C490/A10124, C536/A13086, C536/A6689); the Celma Mastry Ovarian Cancer Foundation ; the Danish Cancer Society (94-222-52); the ELAN Program of the University of Erlangen-Nuremberg; the Eve Appeal; the Helsinki University Central Hospital Research Fund; Helse Vest; Imperial Experimental Cancer Research Centre (C1312/A15589); the Norwegian Cancer Society; the Norwegian Research Council; the Ovarian Cancer Research Fund; Nationaal Kankerplan of Belgium; Grant-in-Aid for the Third Term Comprehensive 10-Year Strategy for Cancer Control from the Ministry of Health Labour and Welfare of Japan; the L & S Milken Foundation; the Polish Ministry of Science and Higher Education (4 PO5C 028 14, 2 PO5A 068 27); Malaysian Ministry of Higher Education (UM.C/HlR/MOHE/06) and Cancer Research Initiatives Foundation; the Roswell Park Cancer Institute Alliance Foundation; the US National Cancer Institute (K07-CA095666, K07-CA143047, K22-CA138563, N01-CN55424, N01-PC067010, N01-PC035137, P01-CA017054, P01-CA087696, P30-CA15083, P50-CA105009, P50- CA136393, R01-CA014089, R01-CA016056, R01-CA017054, R01-CA049449, R01-CA050385, R01-CA054419, R01- CA058598, R01-CA058860, R01-CA061107, R01-CA061132, R01-CA063682, R01-CA064277, R01-CA067262, R01- CA071766, R01-CA074850, R01-CA076016, R01-CA080742, R01-CA080978, R01-CA083918, R01-CA087538, R01- CA092044, R01-095023, R01-CA106414, R01-CA122443, R01-CA112523, R01-CA114343, R01-CA126841, R01- CA136924, R01-CA149429, R03-CA113148, R03-CA115195, R37-CA070867, R37-CA70867, U01-CA069417, U01-CA071966 and Intramural research funds); the US Army Medical Research and Material Command (DAMD17-98-1- 8659, DAMD17-01-1-0729, DAMD17-02-1-0666, DAMD17-02-1-0669, W81XWH-10-1-0280); the National Health and Medical Research Council of Australia (199600 and 400281); the German Federal Ministry of Education and Research of Germany Programme of Clinical Biomedical Research (01 GB 9401); the state of Baden-Württemberg through Medical Faculty of the University of Ulm (P.685); the Minnesota Ovarian Cancer Alliance; the Mayo Foundation; the Fred C. and Katherine B. Andersen Foundation; the Lon V. Smith Foundation (LVS-39420); the Oak Foundation; the OHSU Foundation; the Mermaid I project; the Rudolf-Bartling Foundation; the UK National Institute for Health Research Biomedical Research Centres at the University of Cambridge, Imperial College London, University College Hospital "Womens Health Theme" and the Royal Marsden Hospital; WorkSafeBC.

Supplementary Methods

Summary of data sets

Genetic association analyses were carried out using data from several OCAC genotyping projects. The following numbers are for samples of European ancestry determined using genotype data. These included 2,162 cases and 2,564 controls from a GWAS from North America ("US GWAS"), 1,763 cases and 6,118 controls from a UK-based GWAS ("UK GWAS"), and 441 cases and 442 controls from a second GWAS from North America ("Mayo GWAS") 1-3. 11,030 cases and 21,693 controls from 41 OCAC studies were genotyped using the iCOGS array ("OCAC-iCOGS" stage 1 data). The US and UK GWASs comprised several independent case-control studies, and samples from some of these studies were also subsequently genotyped using the iCOGS array – all duplicates were removed from the analysis. Further details of the component studies are in Supplementary Table 3. Details of genotyping platform used for each data set are shown in Supplementary Table 4. All patient samples were collected with informed patient consent and under the approval of the institutional review board overseeing each study.

Genotype data quality control

UK GWAS: Cases were from four studies (RMH, SEA, UKO and UKR) genotyped using the Illumina 610 Quad array. Controls were blood donor controls and 1958 Birth Cohort controls genotyped on the Illumina 550k array as part of the Welcome Trust Case-Control Consortium. Quality control criteria were applied separately to the case and control sets because they were genotyped separately. SNPs exclusion criteria were: deviation of genotype frequencies from those expected under Hardy-Weinberg equilibrium at P < 10-4, MAF < 1 per cent, MAF between 1 and 5 per cent and call rate < 99 per cent, MAF > 5 per cent and call rate < 95 per cent and test for trend by genotype between the two control sets was significant at P < 10-4. In a union between to two data sets 507,094 SNPs passed QC.

US GWAS: Four case controls studies (MAY, NCO, TBO and TOR) were genotyped using the Illumina 660K array, the NEC case-control study was genotyped using the Illumina 370k and Illumina 317K array and the POL case control study was genotyped using the Illumina 550k array. QC was carried out seperately for the three data sets. SNPs exclusion criteria were: deviation of genotype frequencies from thise expected under Hardy-Weinberg equilibrium at P < 10-5, MAF < 1 per cent, MAF between 1 and 5 per cent and call rate < 99 per cent, MAF > 5 per cent and call rate < 95 per cent. The number of SNPs passing QC was 556,480 (MAY, NEC, NCO, TBO, TOR), 305,690 (NEC/BWH) and 527,435 (POL).

Mayo GWAS: SNPs exclusion criteria were: deviation of genotype frequencies from thise expected under Hardy-Weinberg equilibrium at P < 10-5, fewer than 5 heterozygotes, MAF < 5 per cent and call rate < 99 per cent, MAF > 5 per cent and call rate < 95 per cent. 622568 SNPs failed QC leaving 1587042 for analysis.

COGS: SNPs exclusion criteria were: deviation of genotype frequencies from thise expected under Hardy-Weinberg equilibrium at P < 10-5, fewer than 5 heterozygotes, MAF < 5 per cent and call rate < 99 per cent, MAF > 5 per cent and call rate < 95 per cent. Of 211,155 SNP assays successfully designed and included on the array 199,570 passed QC.

Sample QC

UK GWAS: Exclusion criteria were: conversion rate< 80 per cent ambiguous gender, unresolved genotype duplicates and less than 90 per cent European ancestry using the programme program LAMP4 and reference geneotypes from HapMap (release 22) and subjects. Final sample size was 1,763 cases and 6,118 controls.

US GWAS: Exclusion criteria were: conversion rate < 95 percent, ambiguous gender, unresolved genotype duplicates and less than 80 per cent European ancestry using STRUCTURE. This resulted in a final sample size of 2,162 cases and 2,564 controls.

Mayo GWAS: Exclusion criteria were: samples with a conversion rate of less than 95 percent, samples with heterozygosity >5 standard deviations from the intercontinental ancestry specific mean heterozygosity, samples with ambiguous sex, samples with the lowest call rate from a first-degree

relative pair and samples that were either duplicate samples that were non-concordant for genotype or genotypic duplicates that were not concordant for phenotype. A total of 907 subjects including 453 cases and 454 controls were available for analysis, of which 441 cases and 442 controls were of European ancestry.

COGS: Exclusion criteria were: samples with a conversion rate of less than 95 percent, samples with heterozygosity >5 standard deviations from the intercontinental ancestry specific mean heterozygosity, samples with ambiguous sex, samples with the lowest call rate from a first-degree relative pair and samples that were either duplicate samples that were non-concordant for genotype or genotypic duplicates that were not concordant for phenotype. A total of 44,308 subjects including 18,174 cases and 26,134 controls were available for analysis. Of these 5,556 cases and 1,816 were included in one of the GWAS leaving 12,618 cases and 24,318 controls unique samples. There were 11,030 cases and 21,693 controls of European ancestry.

Imputation.

The number of successfully imputed SNPs (by minor allele frequency) is shown in Supplementary Table 5.

References

1. Permuth-Wey, J. *et al.* LIN28B Polymorphisms Influence Susceptibility to Epithelial Ovarian Cancer. *Cancer Research* **71**, 3896-3903 (2011).
2. Song, H. *et al.* A genome-wide association study identifies a new ovarian cancer susceptibility locus on 9p22.2. *Nat Genet* **41**, 996-1000 (2009).
3. Kuchenbaecker, K.B. *et al.* Identification of six new susceptibility loci for invasive epithelial ovarian cancer. *Nat Genet* **47**, 164-71 (2015).
4. Sankararaman, S., Sridhar, S., Kimmel, G. & Halperin, E. Estimating local ancestry in admixed populations. *Am J Hum Genet* **82**, 290-303 (2008).
